# Mitochondrial Gene Scoring System: Predicting Prognosis and Precision Therapy for TACE Non-Response in Hepatocellular Carcinoma Patients

**DOI:** 10.7150/jca.103946

**Published:** 2025-01-01

**Authors:** Jian-ying Ma, Wei Wei, Yi-xian Wang, Zhen-yu Zhao, Zhen-yu Xiong, Jie Mei, Wen-ze Wu, Jia-wei Guo

**Affiliations:** 1 Department of Interventional, Jingzhou Hospital Affiliated to Yangtze University, Jingzhou, 434020, China.; 2 Department of Immunology, School of Medicine, Yangtze University, Jingzhou 434023, China.; 3 Department of Pharmacology, School of Medicine, Yangtze University, Jingzhou 434023, China.

**Keywords:** hepatocellular carcinoma, mitochondria, transarterial chemoembolization, predictive biomarker, drug sensitivity.

## Abstract

**Objective:** Given the crucial role of mitochondria in the prognosis and treatment of hepatocellular carcinoma (HCC), we aim to develop two independent mitochondrial scoring systems to separately predict patient prognosis and the likelihood of transarterial chemoembolization non-response (TACE NR).

**Methods:** Mitochondria-related candidate genes were selected and analyzed using univariate Cox and LASSO Cox regression analyses to create a risk prognosis score (RPS). Univariate and LASSO logistic regression analyses were used to establish the risk diagnosis score (RDS). Alternative therapies for patients with TACE NR were explored using TIDE and oncoPredict algorithms. The Seurat package was used to study the involvement of the RDS genes in HCC differentiation.

**Results:** The RPS accurately predicts the 1-5 year survival rates of patients with HCC, where higher RPS values were associated with poorer survival outcomes. The RDS model demonstrated a commendable performance in diagnosing TACE NR, as patients with a higher RDS exhibited a greater likelihood of TACE NR. RDS was associated with the infiltration of various immune cells, and patients with lower RDS tended to have higher response rates to immunotherapy and increased sensitivity to JAK1, rapamycin, and AZD2014. By contrast, patients with higher RDS values and a higher probability of TACE NR had more responsive to paclitaxel, dasatinib, and vincristine, suggesting that these drugs are potential alternative therapies. Single-cell sequencing studies have identified ACSM2A as a key player in HCC differentiation and a potential target for therapeutic intervention.

**Conclusion:** The RPS and RDS are important reference points for predicting outcomes and guiding treatment decisions in patients with HCC. Additionally, ACSM2A shows promise as a potential therapeutic target for HCC.

## Introduction

Hepatocellular carcinoma (HCC), the most prevalent form of primary liver cancer, poses a significant global health challenge. It is the sixth most common cancer and the fourth leading cause of cancer-related deaths worldwide 1. The incidence of HCC is increasing, primarily driven by chronic liver diseases, such as hepatitis B and hepatitis C virus infections, excessive alcohol consumption, and the increasing prevalence of non-alcoholic fatty liver disease. It is closely linked to metabolic syndrome and diabetes mellitus 2. Despite advancements in medical treatment and surgical procedures, the prognosis of patients with HCC remains poor, largely due to delayed detection and the aggressive nature of the disease. Transarterial chemoembolization (TACE) is commonly used as the primary treatment for patients with intermediate-stage HCC who are unsuitable candidates for surgery or liver transplantation. TACE administers chemotherapy directly to the tumor site while obstructing the blood supply, thereby intensifying the localized impact of the drug and reducing systemic exposure 3. Although TACE has demonstrated effectiveness in prolonging survival and managing tumor growth in numerous cases, a notable proportion of patients develop resistance to this treatment, leading to unfavorable outcomes. Roughly 20-30% of patients with HCC do not exhibit a positive response to initial TACE therapy, emphasizing the necessity for alternative treatment approaches 4. TACE non-response (NR) in certain patients remains a significant obstacle, diminishing the overall efficacy of this therapeutic option.

Given the poor prognosis of HCC and the varied responses observed in patients undergoing TACE, there is a pressing need for models that can predict patient outcomes and identify individuals who are unlikely to benefit from TACE. Accurately predicting the treatment response has the potential to greatly enhance clinical decision-making, enabling the development of more tailored and efficacious treatment strategies. Therefore, it is imperative to develop robust predictive models that can predict outcomes and inform therapeutic choices. Moreover, exploring alternative therapies for patients with TACE NR is crucial for improving survival rates and quality of life. Research into these alternatives is vital as it may open new avenues for therapy and provide hope for patients who do not benefit from standard approaches.

Mitochondria are central to the pathogenesis and progression of HCC, influencing TACE response 5. Mitochondrial dysfunction is a key characteristic of cancer and leads to changes in energy metabolism, resistance to cell death, and increased production of reactive oxygen species. These abnormalities support the survival and growth of cancer cells, and underscore the significance of mitochondria in cancer biology. Research has indicated that mutations in mitochondrial DNA and variations in mitochondrial gene expression have been linked to the development of HCC and poor treatment outcomes 6. The intricate network of mitochondria-related pathways in cancer cells suggests that these pathways may serve as potential targets for therapeutic strategies 7. The modulation of mitochondrial function may improve the efficacy of TACE by increasing the sensitivity of HCC cells to treatment, potentially leading to better patient outcomes.

Considering the critical role of mitochondria in HCC, we utilized machine learning algorithms to identify mitochondria-related genes and developed two distinct gene expression scores. These scores aim to predict the prognosis of patients with HCC and identify those who may not respond well to TACE. Furthermore, machine learning algorithms have been used to investigate alternative therapies for TACE-resistant patients, with the goal of enhancing personalized treatment approaches and clinical outcomes. The integration of machine learning in this context marks a significant advancement, offering the potential to unveil intricate patterns and relationships that may not be discernible using traditional analytical methods. The establishment of these scores not only helps in predicting prognosis and treatment response, but also offers insights into the underlying mechanisms of treatment response, guiding future research and therapeutic innovations.

## Materials and Methods

### Data collection

Sequencing data for HCC were obtained from the TCGA (LIHC), ICGC (LIRI-JP), and GEO databases (GSE104580 and GSE242889). Immunotherapy sequencing data were obtained from the GSE78220 dataset. Immune checkpoint genes have been identified previously 8. Mutation data specific to HCC were extracted from the TCGA database, while human mitochondrial genes were sourced from MitoCarta 3.0. The specific criteria for patient inclusion in our study were as follows: Our study exclusively included patients with HCC and excluded those with other liver cancer subtypes. Additionally, individuals with a survival time of less than 30 days were excluded from our analysis because of the potential skewing effect of severe conditions or complicating factors on the model predictions.

### Weighted gene co-expression network analysis

Weighted gene co-expression network analysis (WGCNA) was conducted on the dataset GSE104580. A soft-thresholding power of 4 was selected to establish a scale-free network topology. Initial analysis identified multiple modules of co-expressed genes, in which the blue and brown modules show the strongest correlation with TACE NR. These modules were for further investigation. Visualization and quantification of the connections between the modules and the TACE NR trait helped identify potentially important biological pathways and gene signatures.

### Identification of differentially expressed genes

Differential analysis was performed on the TCGA-LIHC dataset using the limma package to identify genes that differentially expressed between HCC and adjacent normal tissues. Additionally, analysis was conducted on samples with high and low risk diagnosis score (RDS) to distinguish the genes that were differentially expressed between high RDS and low RDS. The criteria for differentially expressed genes (DEGs) included an absolute log2 fold change greater than 1 and an adjusted p-value less than 0.05.

### Identification of differentially expressed mitochondrial-related genes

DEGs were derived from a differential expression analysis of TCGA-LIHC, human mitochondrial genes were obtained from the MitoCarta 3.0, and blue and brown module genes correlated with TACE NR were identified through WGCNA analysis. The intersection of these sets allowed the identification of mitochondria-related DEGs for further refinement using machine learning techniques.

### Selection of screening algorithm

To construct the RPS, we employed univariate Cox regression followed by LASSO Cox regression, as these methods are highly effective in managing high-dimensional genomic data. Univariate Cox regression allows for the initial identification of genes with significant prognostic impact, while LASSO Cox regression addresses the potential issue of multicollinearity by applying regularization. LASSO's ability to perform variable selection ensures that only the most relevant prognostic genes are included, thereby improving interpretability and robustness in predicting patient outcomes in HCC. This stepwise approach not only refines the gene selection process but also prevents overfitting, enhancing the model's predictive accuracy and clinical applicability. For the RDS, we adopted a similar approach by first using univariate logistic regression to identify genes associated with TACE NR. We then applied LASSO logistic regression to select the most predictive features while controlling for multicollinearity. The strength of this approach lies in its ability to maintain model simplicity without sacrificing accuracy, ensuring that the RDS model remains both interpretable and highly effective in clinical decision-making, particularly for identifying patients likely to exhibit resistance to TACE therapy.

### Construction of a mitochondrial gene-associated risk prognostic score

Mitochondria-related DEGs were analyzed using univariate and LASSO Cox regression analyses to identify the genes associated with the risk prognosis score (RPS). The RPS of each patient was calculated using the following formula:



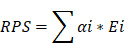



αi represents the coefficient of gene i in the RPS, and Ei represents the expression level of gene i in a patient. Patients were divided into high- and low- RPS groups based on the median RPS in the machine learning cohorts (training, test, and validation sets). The predictive ability of RPS for prognosis was assessed using Kaplan-Meier (KM) and receiver operating characteristic (ROC) curves.

### Construction of a mitochondrial gene-associated risk diagnosis score

Mitochondria-related DEGs were analyzed using a combination of univariate and LASSO logistic regression to identify genes linked to the RDS, with TACE NR as the endpoint event. The RDS for each patient was calculated using the following formula:



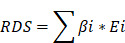



βi represents the contribution coefficient of gene i in the RDS, and Ei represents the expression level of gene i in a patient. The patients were divided into high- and low- RDS groups based on the median RDS value in the machine learning cohorts, such as the training and test sets.

### Construction of nomogram

This study analyzed the RPS and clinical characteristics of patients with TCGA-LIHC, such as age (categorized as old if > 65 years, otherwise young), sex, stage, T-stage, N-stage, and M-stage. Initially, a univariate Cox regression analysis was conducted to identify the characteristics that impact prognosis, with those having a p-value less than 0.05, progressing to a multivariate Cox regression analysis to assess their significance in the presence of multiple variables. This method aims to isolate independent prognostic features by eliminating confounding effects. These features were then used to develop a nomogram for a risk prognostic model, which was evaluated using area under the curve (AUC) values, calibration curves, C-index, and decision curves. In the absence of clinical data in the GSE104580 dataset, a risk diagnostic model was created solely from the RDS, followed by the construction of a nomogram. The diagnostic efficiency of the risk diagnosis model was assessed using ROC, calibration, and decision curves.

### Exploration of the tumor microenvironment

In our study, we utilized the IOBR package to apply six cell infiltration algorithms for investigating the tumor microenvironment in patients. These algorithms included xCell, quantiseq, EPIC, TIMER, MCPcounter, and CIBERSORT.

### Gene Ontology and Kyoto Encyclopedia of Genes and Genomes enrichment analyses

This study utilized Gene Ontology (GO) and Kyoto Encyclopedia of Genes and Genomes (KEGG) enrichment analyses were used to explore the functions of DEGs between high and low RDS. An adjusted p-value of less than 0.05 was used as the screening criterion.

### Mutation analysis

The maftools package was utilized to generate waterfall plots illustrating the mutation landscape in LIHC patients categorized by high and low RDS.

### Predicting treatment sensitivity in patients with hepatocellular carcinoma

This study examined the predictive potential of RDS for immunotherapy and drug sensitivity. The TIDE algorithm was used to assess immune therapy response and TIDE scores in patients from the LIHC, LIRI-JP, and GSE104580 datasets. The oncoPredict package was used to calculate the half maximal inhibitory concentration (IC50) values for 198 targeted/chemotherapy drugs for every patient in these datasets. Drug target information was obtained from the DrugBank database.

### Single-cell RNA sequencing data analysis

The Seurat package was utilized to analyze the HCC single-cell dataset GSE242889. Initially, single-cell data were filtered to exclude low-quality cells based on the following specific criteria: feature count range of 500-4200, UMI count range of 1700-24000, mitochondrial gene percentage below 40%, hemoglobin gene percentage below 0.1%, and ribosomal gene percentage below 30%. The subsequent steps involved removing the influence of the cell cycle, eliminating batch effects using the harmony package, and following the standard Seurat analysis workflow for dimensionality reduction, clustering, and visualization. The CellChat package was used to investigate interactions between different cell types, while the copycat package was applied to distinguish HCC cells from normal hepatocytes. Lastly, the slingshot package was utilized to analyze the differentiation trajectory of hepatocytes.

### Cell lines and culture

The L02, Hep3B, and Huh7 cells were purchased from the Type Culture Collection of the Chinese Academy of Sciences (Shanghai, China). All used cell lines were maintained in Dulbecco's Modified Eagle Medium supplemented with 10% fetal bovine serum and 1% penicillin-streptomycin and grown in a humidified atmosphere containing 5% CO2 at 37 °C. All cell lines were authenticated using short tandem repeat (STR) genotyping and tested negative for Mycoplasma.

### Quantitative real-time PCR

mRNA levels were analyzed by qPCR, as previously described 9-11. Briefly, total RNA was extracted from cells using TriPure Isolation Reagent (Roche, Basel, Switzerland) according the manufacturer's guidelines. The extracted RNA was then reverse-transcribed into cDNA using a Transcriptor First Strand cDNA Synthesis Kit (Roche) with 2 µg of total RNA. Real-time PCR was conducted on a MyiQ Single Color Real-time PCR Detection System (Bio-Rad Laboratories, Hercules, CA, USA) using SYBR Green PCR Master Mix (Bio-Rad Laboratories). The mRNA expression levels were normalized to β-actin using the comparative 2-ΔΔCT method. Primer sequences for the specified genes are listed in Table 1.

### Statistical analysis

All statistical analyses were performed using R software (version 4.3.1). Differences between the two groups were analyzed using the Wilcoxon test, chi-square test, or Fisher's exact test. Spearman's correlation analysis was used to assess correlations between variables. Statistical significance was set at p < 0.05.

## Results

### Workflow of this study

**Figure 1:**
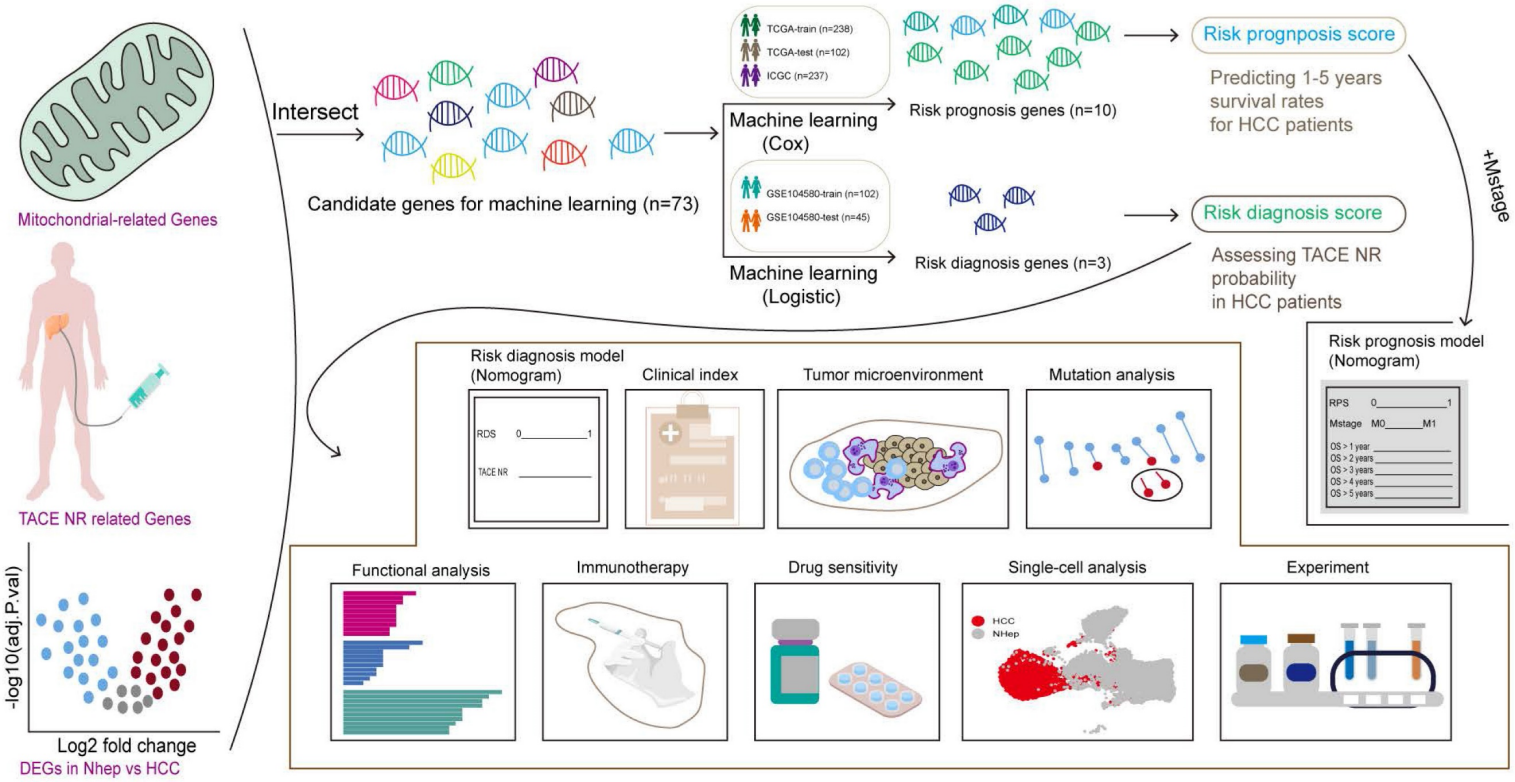
The flowchart graph of this study.

The workflow of this study is illustrated in Figure 1.

### Patient data

To prevent overfitting in our model, we randomly divided the patient data in a 7:3 ratio, with 70% allocated to the training set and the remaining 30% to the test set. The specifics of the split patient data are presented in Tables 2 and 3.

### Acquisition of differentially expressed mitochondrial genes

**Figure 2:**
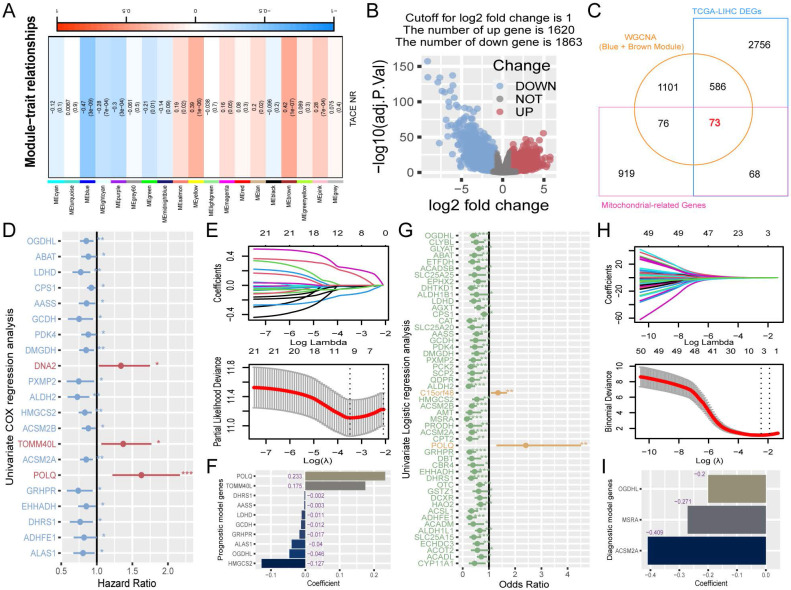
Construction of RPS and RDS. (A) Relationship between gene modules and TACE NR. (B) Volcano plot of DEGs between HCC and normal tissues. (C) Venn diagram of the intersection among DEGs in HCC and normal tissues, mitochondrial genes, and TACE NR-associated module genes. (D) Univariate Cox regression analysis identified 21 genes associated with HCC prognosis. (E) LASSO Cox regression analysis ultimately selected 10 genes for constructing RPS. (F) Contribution coefficients of RPS-related genes in RPS. (G) Univariate logistic regression analysis identified 51 genes associated with TACE NR. (H) LASSO logistic regression analysis ultimately selected 3 genes for constructing RDS. (I) Contribution coefficients of RDS-related genes in RDS.

To identify candidate genes for machine learning screening, the genes must have met three criteria: association with TACE NR, differential expression in HCC, and mitochondrial classification. Using WGCNA, we explored the module genes associated with TACE NR. Initially, clustering of GSE104580 patients and setting the height threshold at 130 identified one outlier patient who was excluded (Figure S1A). Co-expression modules were constructed with the remaining 146 patients, revealing that the blue and brown modules were highly correlated with TACE NR (Figure 2A). Differential expression analysis of the TCGA-LIHC dataset identified 3483 DEGs between HCC and normal tissues (Figure 2B). Extracting 1136 human mitochondrial genes from the MitoCarta 3.0 database, we identified 73 differentially expressed mitochondrial genes as candidates for machine learning screening by intersecting TACE NR-associated genes, TCGA-LIHC DEGs, and mitochondrial genes (Figure 2C).

### Construction of a risk prognosis score using prognosis-associated differentially expressed mitochondrial genes

Initially, a univariate Cox regression analysis was conducted on 73 mitochondria-related DEGs from the TCGA-trained HCC data, highlighting 21 genes with a significant prognostic influence (p < 0.05) (Figure 2D). To refine the model features and address multicollinearity, LASSO Cox regression analysis was performed on these 21 genes (Figure 2E), which resulted in the identification of 10 genes closely linked to prognosis (Figure 2F). The RPS for each patient was calculated based on the contribution coefficients of the ten prognostic genes and their respective expression levels. The formula is as follows: RPS = (POLQ * 0.233) + (TOMM40L * 0.175) + (DHRS1 * -0.002) + (AASS * -0.003) + (LDHD * -0.011) + (GCDH * -0.012) + (GRHPR * -0.017) + (ALAS1 * -0.040) + (OGDHL * -0.046) + (HMGCS2 * -0.127). To enhance clarity, the RPS values were normalized to the range of 0-1.

### Construction of a risk diagnosis score using diagnosis-associated differentially expressed mitochondrial genes

Utilizing the TACE treatment response data from the GSE104580-train, we performed univariate logistic regression analysis on 73 mitochondrial-related DEGs, revealing 51 genes that were significantly associated with the diagnosis of TACE NR (p < 0.05) (Figure 2G). After further refining the model, LASSO logistic regression analysis identified three risk diagnostic genes that were more closely associated with TACE NR (Figure 2H, I). Subsequently, the RDS for each patient was computed using the following formula: RDS = (OGDHL * -0.200) + (MSRA * -0.271) + (ACSM2A * -0.409). The RDS was then normalized to a range of 0-1.

### Evaluation of the performance of the risk prognosis score

**Figure 3:**
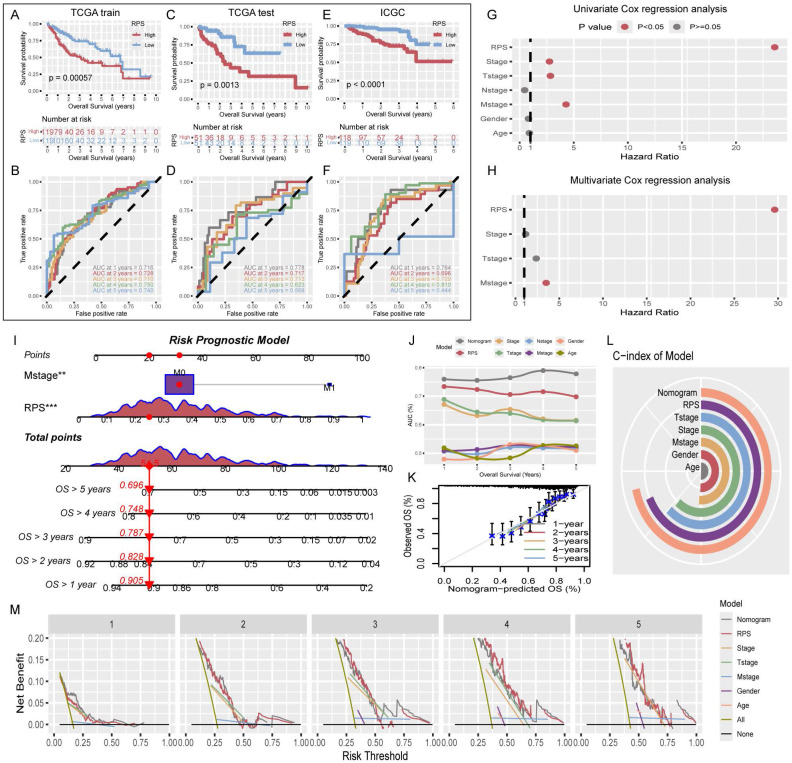
Evaluation of RPS and construction of a risk prognosis model. (A) KM survival curves for overall survival (OS) of high- and low- RPS groups. (B) ROC curve predicting 1-5 year OS of patients with HCC using RPS. (C-F) Test cohort TCGA-test and external validation cohort ICGC. (G) Univariate Cox regression analysis for the clinicopathologic characteristics and RPS. (H) Multivariate Cox regression analysis for the clinicopathologic characteristics and RPS. (I) The nomogram of the risk prognosis model. (J) Comparison of 1-5 year AUC values among different models. (K) Calibration plots showing the probability of 1-5 year OS. (L) Comparison of C-index values among different models. (M) Comparison of decision curves between different models.

The RPS was calculated for TCGA-train, TCGA-test, and ICGC patients, who were then categorized into high- and low- RPS groups based on the median. In the TCGA-train cohort, KM analysis revealed that patients with a high RPS had a worse prognosis than those with a low RPS (Figure 3A). The ROC curve showed that RPS accurately predicted the 1- to 5-year prognosis with AUC values above 0.7, indicating good model performance in the training set (Figure 3B). Internal validation using the test cohort demonstrated results consistent with the training set, showing good classification and accurate predictions for 1- to 4-year prognosis, with a slight decrease in accuracy in the fifth year (Figure 3C, D). Furthermore, external validation using the ICGC dataset also yielded satisfactory KM results (Figure 3E), with the ROC curve showing good predictive performance for 1- to 4-year survival, but suboptimal performance in the fifth year with an AUC less than 0.5 (Figure 3F).

### Construction and evaluation of the risk prognostic model

To enhance the predictive power and clinical relevance of the RPS, clinical indicators that independently affect prognosis were incorporated into a comprehensive risk prognostic model. Univariate Cox regression analysis of RPS and relevant clinical indicators among all TCGA patients revealed statistically significant differences in the prognosis of RPS, Stage, T-stage, and M-stage (Figure 3G). Subsequently, multivariate Cox regression analysis incorporating these four factors identified RPS and M-stage as independent prognostic indicators (Figure 3H). Using the RPS and M-stage, we constructed a risk prognostic model and generated a nomogram (Figure 3I). Evaluation of the risk prognostic model demonstrated superior performance in terms of AUC values for 1-5 year compared with individual prognostic indicators. Our findings underscore the superior predictive ability of the RPS over traditional clinical staging indicators (Figure 3J). Furthermore, we compared our model with those developed by other researchers. The comparison of AUC values at 1, 3, and 5 years demonstrated superior performance of our model (Figure S2) 12-17. Additionally, calibration curves, C-indices, and decision curves collectively affirmed the accuracy of the risk prognostic model (Figure 3K-M).

### Construction and validation of the risk diagnosis model

**Figure 4:**
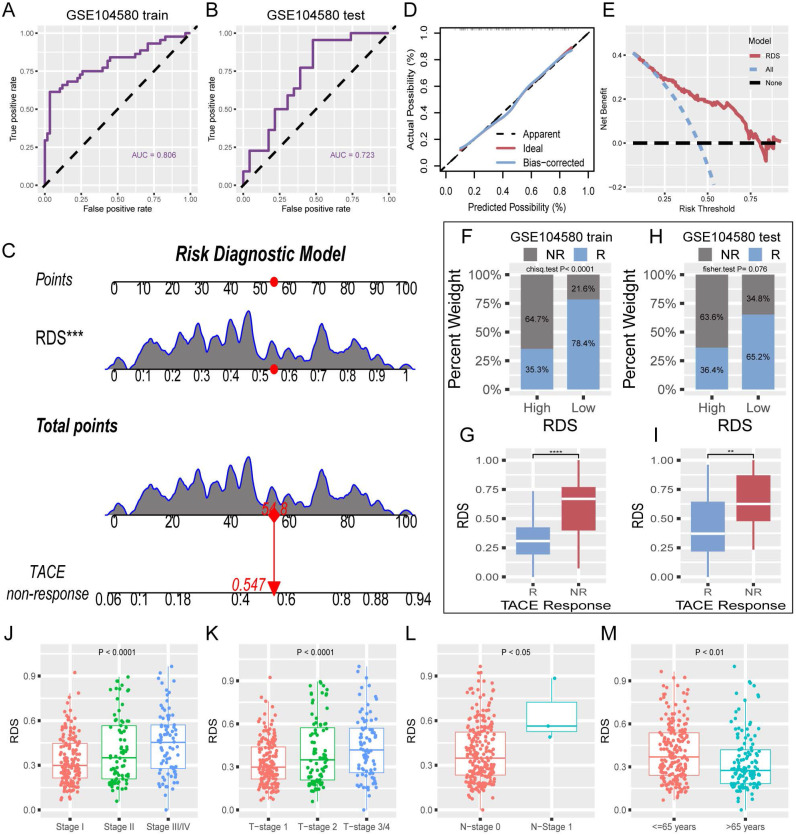
Evaluation of RDS and construction of a risk diagnosis model. (A) The ROC curve of the training cohort reveals that RDS excels in diagnosing TACE NR, (B) with similarly commendable performance in the test cohort. (C) The nomogram of the risk diagnosis model. (D) Calibration curve of the risk diagnostic model. (E) Decision curve of the risk diagnostic model. (F-I) The training cohort results indicate that the high RDS group exhibits a higher probability of TACE NR, and patients in the NR group have elevated RDS. The test cohort displays a consistent trend. (J-M) Distribution differences of RDS in clinicopathologic characteristics.

The absence of clinical data in GSE104580 led to the development of a risk diagnostic model that essentially functions as a RDS. The results from the training set indicated that this model exhibited strong diagnostic efficacy, with an AUC exceeding 0.8 (Figure 4A). Similarly, the test set displayed favorable diagnostic outcomes, with an AUC surpassing 0.7 (Figure 4B). A nomogram illustrating the risk diagnostic model was constructed (Figure 4C). Both calibration and decision curves confirmed the robust diagnostic performance of the model (Figure 4D, E). By categorizing the patients in the training and test sets into high- and low- RDS groups based on the median RDS, we investigated the effectiveness of RDS in diagnosing TACE NR. In the training set, patients with a low RDS exhibited a significantly lower TACE NR rate (21.6%) than those with a high RDS (64.7%) (Figure 4F). Box plots further illustrated that TACE NR patients had a higher RDS (Figure 4G). This trend was consistent in the test set (Figure 4H, I). Analysis of TCGA-train+test dataset revealed a progressive increase in RDS with advancing clinical stage (Figure 4J-L). Intriguingly, patients aged 65 and under displayed higher RDS levels compared to those over 65 (Figure 4M).

### Differences in tumor microenvironment infiltration among patients with different transarterial chemoembolization responses

**Figure 5:**
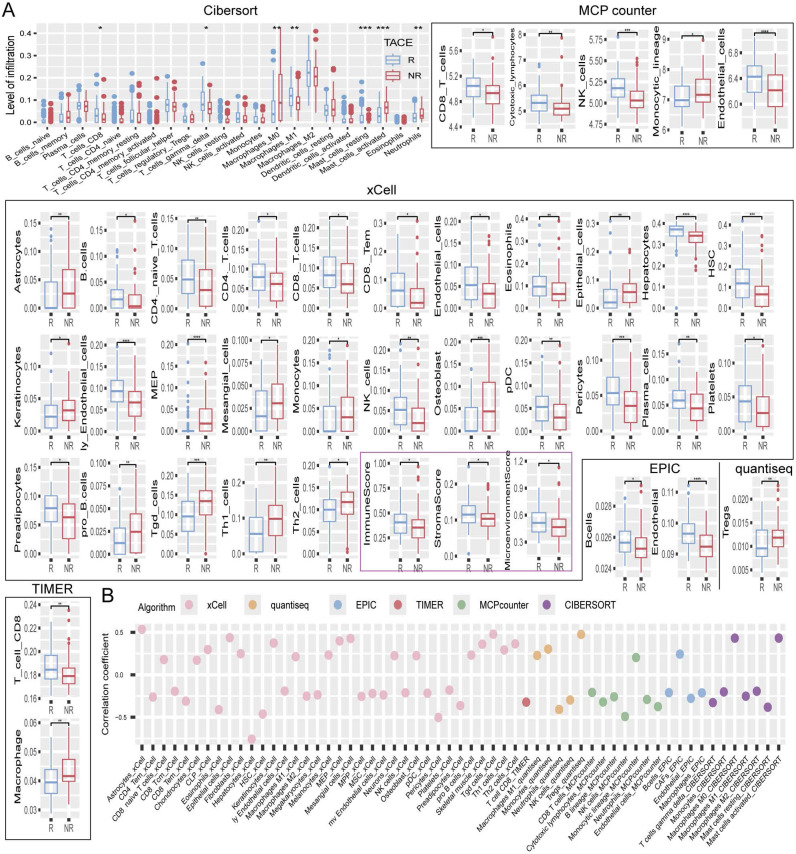
Tumor microenvironment. (A) Differences in the levels of tumor microenvironment cell infiltration between TACE R and NR groups. (B) Bubble chart of the correlation between RDS and tumor microenvironment cell infiltration levels.

The TACE responsive (R) group consistently exhibited higher levels of CD8+ T and NK cells across various analysis algorithms, such as CIBERSORT, MCPcounter, xCell, EPIC, quantiseq, and TIMER, indicating the robust presence of these cytotoxic immune cells. Moreover, M1 macrophages, known for their pro-inflammatory and anti-tumor properties 18, 19, were significantly more abundant in the R group, whereas dendritic cells, which are essential for antigen presentation and immune response initiation 20, also showed increased infiltration levels. B cells were more prevalent in the R group, reflecting an enhanced humoral immune response. In contrast, the NR group displayed lower levels of these key immune cells, suggesting a less active immune microenvironment (Figure 5A). Further analysis revealed a negative correlation between RDS and T cells as well as NK cells, indicating that a higher RDS is linked to lower infiltration levels of these cytotoxic cells. Monocytes and M1 macrophages also showed a negative correlation with RDS, whereas M0 macrophages and activated mast cells exhibited a positive correlation, indicating that an increase in RDS leads to a shift towards a more immunosuppressive and inflammatory tumor microenvironment. Interestingly, RDS was strongly negatively correlated with hepatocyte count, suggesting a significant decrease in their number as RDS increased (Figure 5B).

### Functional enrichment analysis of differentially expressed genes in high and low risk diagnosis score

Differential expression analysis conducted on the high- and low- RDS groups revealed 362 DEGs (Figure S3A). Subsequently, enrichment analysis was performed to investigate functional differences between the two groups. GO and KEGG enrichment analyses revealed significant variations in the metabolic and detoxification processes. Notably, the tumor-related functions encompassed small-molecule catabolic processes, cellular responses to xenobiotic stimuli, oxidoreductase activity, and heme binding (Figure S3B). The enriched pathways included complement and coagulation cascades, PPAR signaling, cytochrome P450-mediated drug metabolism, and fatty acid degradation (Figure S3C).

### Mutational landscape between the high and low risk diagnostic score

This study presents a mutation waterfall plot comparing high and low RDS, as shown in Figure S4A, B. These findings indicate that in both the TCGA-train and test datasets, CTNNB1 had the highest mutation rate in low- RDS samples, whereas TP53 was more prevalent in high- RDS samples.

### Application value of the risk diagnosis score in immunotherapy

**Figure 6:**
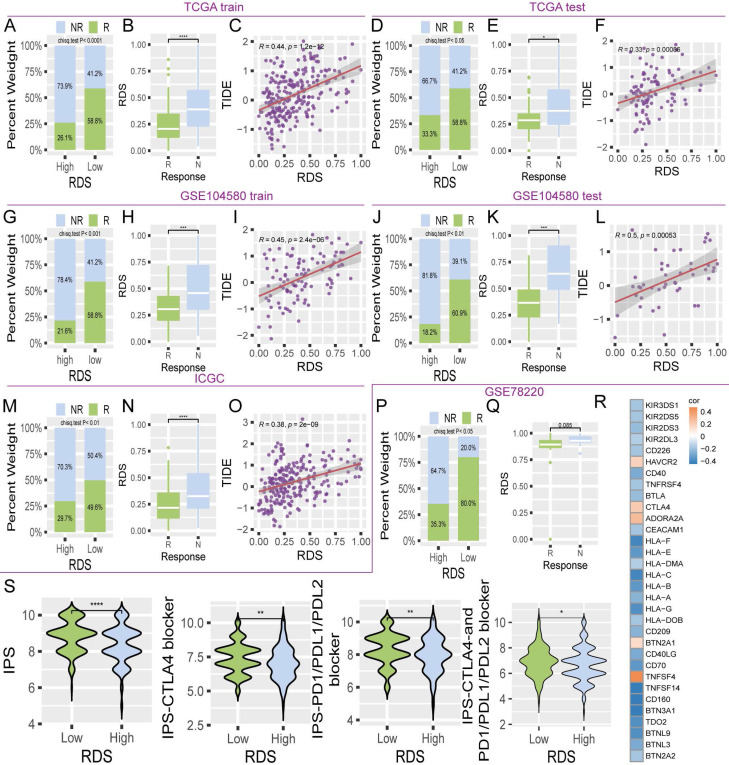
The value of RDS in immunotherapy. (A-O) The bar chart indicates that patients with low RDS are more sensitive to immunotherapy, with the immunotherapy response group exhibiting lower RDS. The scatter plot demonstrates a positive correlation between RDS and TIDE. The results show a consistent trend across multiple cohorts. (P, Q) In the GSE78220 immunotherapy cohort, it is also evident that lower RDS is associated with a higher immunotherapy response, with patients responding to immunotherapy exhibiting a lower RDS. (R) Heatmap of the correlation between RDS and immune checkpoints. (S) Differences in IPS between high and low RDS groups.

Immunotherapy for HCC has shown significant promise in leveraging the patient's immune system to target and eliminate cancer cells, ultimately improving the prognosis 21. This study assessed the utility of the RDS in immunotherapy. The TIDE algorithm consistently revealed that individuals with a low RDS had a higher response rate to immunotherapy across multiple datasets (TCGA, GSE104580, and ICGC), whereas those who did not respond well to immunotherapy had higher RDS levels. Additionally, RDS positively correlated with TIDE scores (Figure 6A-O). Validation using the external dataset GSE78220 further confirmed that a low RDS was correlated with a better response rate, whereas unresponsive patients had higher RDS levels (Figure 6P, Q). Correlation analyses indicated a negative relationship between RDS and various immune checkpoints (Figure 6R). Notably, this study also observed that the immunophenoscore (IPS) was higher in patients with a low RDS than in those with a high RDS, both in the absence of immune checkpoint inhibitors and when inhibitors targeting CTLA4, PD1, PDL1, and PDL2 were used (Figure 6S). Collectively, these findings suggest that individuals with a low RDS tend to exhibit more favorable responses to immunotherapy than those with a high RDS.

### Effective therapeutic drugs for the high and low risk diagnosis score

**Figure 7:**
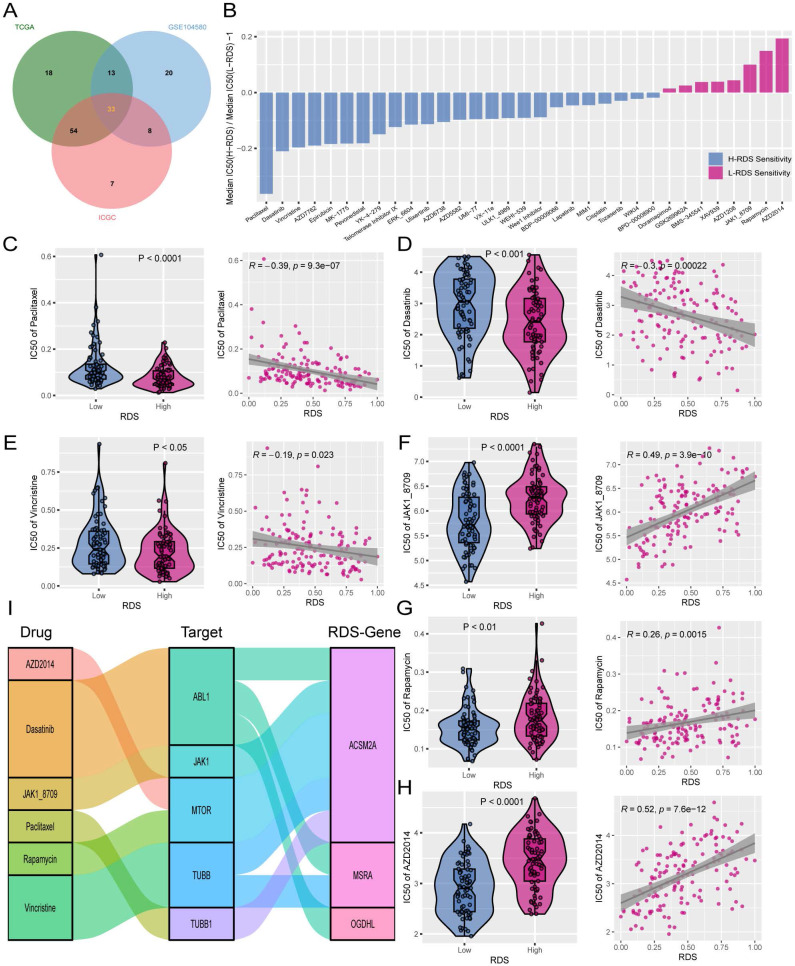
Exploration of potential therapeutic drugs for patients with high and low RDS. (A) A Venn diagram showing the intersection of significant drugs among the three cohorts. (B) The normalized IC50 values of 33 drugs between high and low RDS ratios. (C-E) High RDS is more sensitive to paclitaxel, dasatinib, and vincristine, with a negative correlation between RDS and their IC50 values. (F-H) Low RDS is more sensitive to JAK1, rapamycin, and AZD2014, with a positive correlation between RDS and their IC50 values. (I) The correlation between RDS genes and drug therapeutic targets.

To identify alternative therapies for a high RDS and explore additional treatment options for a low RDS, machine learning algorithms were used to predict the IC50 values of 198 targeted/chemotherapy drugs in patients from the TCGA, GSE104580, and ICGC cohorts. This study focused on drugs that showed significant IC50 differences between high and low RDS. A total of 33 candidate drugs were identified with statistically significant intersections across all three datasets (Figure 7A). Sensitivity to these drugs decreased in the high- RDS group and increased in those with a low RDS in a sequential manner (Figure 7B). From these, six drugs were selected as potential clinical treatments for HCC. A high- RDS was found to be more sensitive to paclitaxel, dasatinib, and vincristine, with RDS showing a negative correlation with the IC50 values of these drugs (Figure 7C-E). Conversely, JAK1, rapamycin, and AZD2014 were more effective in patients with a low RDS, with RDS showing a positive correlation with the IC50 values of these drugs (Figure 7F-H). Further analysis revealed that the targets of these six drugs were linked to RDS genes, with ACSM2A showing correlation with multiple drug targets (Figure 7I).

### Establishment of a hepatocellular carcinoma atlas using single-cell sequencing

**Figure 8:**
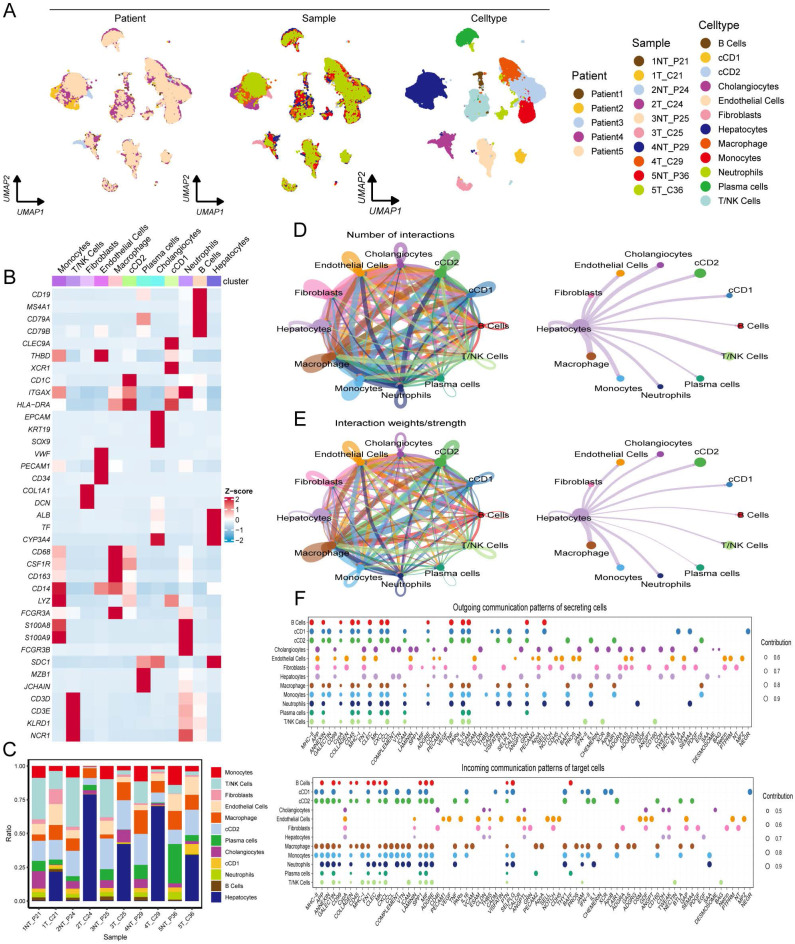
Construct a HCC atlas using single-cell sequencing data. (A) Clustering and annotation of single-cell RNA sequencing data in GSE242889. (B) The heatmap illustrates the expression patterns of markers across various cell types. (C) The proportional bar chart depicts the distribution of cells across different samples. (D, E) The number of interactions and the interaction weights/strengths between cells in the tumor microenvironment of HCC. (F) The bubble plots indicate the key outgoing and incoming signaling patterns of the cell, respectively. The size of the dots is proportional to the calculated contribution fraction in cellular communication, with higher contribution fractions representing signaling pathways that are more abundant in cellular interactions.

Single-cell sequencing can reveal differences in gene expression at the individual cell level, shedding light on the cellular heterogeneity and functions of rare cell populations. This motivated the further exploration of RDS-related genes using single-cell sequencing. A comprehensive single-cell atlas of HCC was generated by analyzing single-cell data and employing classical markers to categorize cell populations. This atlas includes a variety of cell types such as B cells, classical dendritic cell types 1 and 2 (cDC1 and cDC2), cholangiocytes, endothelial cells, fibroblasts, hepatocytes, macrophages, monocytes, neutrophils, plasma cells, and T/NK cells (Figure 8A). The heatmap illustrates the expression levels of specific markers across different cell types (Figure 8B), and the proportional bar chart displays the distribution of cells in the various samples (Figure 8C). The tumor microenvironment is characterized by a dynamic interplay between tumor cells, immune cells, and stromal cells, which collectively influence tumor fate. Our findings regarding cell communication revealed interactions between diverse cell types. Notably, we observed that hepatocytes engaged in robust communication with macrophages and monocytes compared to other cell types (Figure 8D, E). Subsequently, we analyzed the input and output signals of different cell types in the HCC tumor microenvironment. Various cells are capable of producing and releasing a variety of cytokines or ligands as signaling molecules while also responding to external ligands through receptors. The key signals facilitating communication between different cell types are shown (Figure 8F). Overall, ligand-receptor interactions among diverse cell types within the tumor microenvironment emphasize the importance of each cellular subset in tumor development.

### Identification of dynamic risk diagnostic score genes during the transition of hepatocyte states

**Figure 9:**
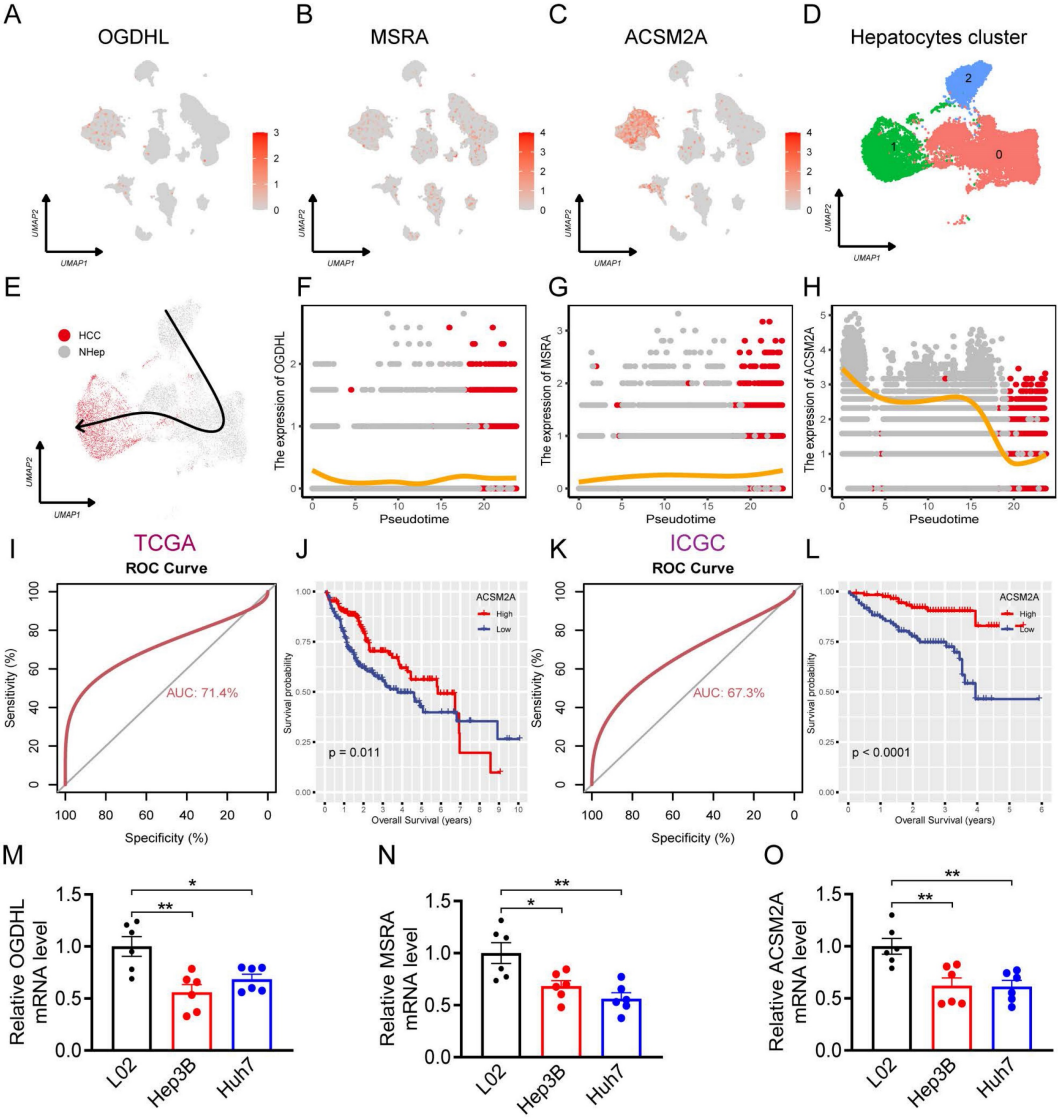
The role of RDS in hepatocyte differentiation. (A-C) Feature plots showing the distribution and expression levels of the RDS genes. (D) UMAP plot of hepatocyte reclustering. (E) Trajectory plot of hepatocyte differentiation. (F-H) Expression changes of the RDS gene during hepatocyte differentiation. (I-L) The ROC curve results indicate that ACSM2A is effective in diagnosing HCC, while the KM survival curve shows that patients with high ACSM2A expression have a favorable prognosis. (M-O) The mRNA levels of OGDHL, MSRA, and ACSM2A in L02 normal liver and Hep3B and Huh7 liver cancer cells.

The expression levels of RDS genes in various cell types within the HCC single-cell atlas were determined. The findings showed that OGDHL was mainly expressed in hepatocytes (Figure 9A), MSRA was expressed in various cell types (Figure 9B), and ACSM2A was particularly expressed in hepatocytes (Figure 9C). Subsequently, hepatocytes in the HCC single-cell atlas were re-clustered, revealing three distinct subgroups (Figure 9D). Using the copycat package, we determined that subgroup 1 comprised of HCC cells, whereas subgroups 0 and 2 represented normal hepatocytes. Trajectory analysis using the slingshot package illustrated the differentiation processes of these cell subgroups (Figure 9E). Upon tracking the expression changes in RDS genes over pseudotime, we noticed slight variations in OGDHL and MSRA expression during the differentiation of hepatocytes into HCC cells, suggesting their potential involvement in this process (Figure 9F, G). Interestingly, ACSM2A expression decreased gradually with pseudotime and significantly decreased as the hepatocytes transitioned into HCC cells (Figure 9H). This implies a crucial role for ACSM2A in determining the fate of hepatocytes transitioning to HCC. The diagnostic ROC curve and KM plot supported the association between ACSM2A expression and the diagnosis and prognosis of HCC, further emphasizing its importance in the disease (Figure 9I-L). Finally, we validated the expression of the RDS genes at the cellular level and found that all three genes were downregulated in HCC cells (Figure 9M-O).

## Discussion

In this study, machine learning algorithms were used to identify key mitochondrial genes and develop two independent scoring systems. One of these, the RPS, was employed to forecast the prognosis of patients over 1-5 year. In the TCGA dataset, RPS exhibited exceptional performance in predicting the 1-5 year prognosis of patients with HCC, with consistently high AUC values across all time points, indicating strong predictive accuracy. However, upon independent validation using the ICGC dataset, the AUC value for the fifth year fell below 0.5, indicating a sub-optimal outcome. Subsequent analysis suggested that this outcome was likely influenced by sample bias, as only two patients with HCC in the ICGC dataset survived beyond five years, leading to a substantial error in the fifth-year prediction. Despite this limitation, the RPS remains clinically valuable in predicting the prognosis of patients with HCC. Stratification of patients into high- and low- RPS groups based on median RPS values revealed significantly poorer prognoses in the high- RPS group, emphasizing the need for increased attention and intervention in these patients to enhance their prognosis. Furthermore, the integration of clinical characteristics into a risk prognosis model alongside the RPS improved predictive accuracy and clinical utility.

TACE NR has become a significant challenge in HCC treatment. The RDS was developed to identify patients with TACE NR. TACE NR is typically defined as tumor progression after TACE treatment. While previous studies have focused on radiological characteristics or common tumor markers to predict TACE NR 22, our study is the first to explore the role of mitochondrial genes as biomarkers of TACE NR. By analyzing the expression levels of the three RDS genes, our method accurately predicted TACE NR probability, assisting clinicians in making treatment decisions and maximizing clinical benefits. Our results showed that the RDS performed well in diagnosing TACE NR, with an AUC value exceeding 0.7. The patients were categorized into high- and low- RDS groups based on the median, with the high- RDS group having a higher risk of TACE NR. Therefore, RDS not only serves as a valuable diagnostic tool for TACE NR but also provides essential guidance for treatment decisions.

In practice, the RPS and RDS systems are well-suited for integration into clinical workflows, leveraging gene expression data that can be easily obtained through next-generation sequencing (NGS), a technology increasingly adopted in hospitals for cancer diagnostics and therapeutic decision-making. As NGS becomes more widely available and its costs continue to decrease, these models become increasingly feasible for routine clinical application. Moreover, to enhance their scalability and accessibility in resource-limited settings, integrating simpler clinical markers, such as blood-based biomarkers, could further broaden their utility. The RPS and RDS systems can be computed directly from existing genomic data, allowing for seamless incorporation into current clinical platforms. This enables real-time, automated scoring that provides actionable prognostic and therapeutic insights, improving precision in patient management. Combining NGS data with readily available, cost-effective biomarkers could offer a more practical and scalable solution, making these systems widely applicable across diverse healthcare environments without disrupting established workflows.

RPS-related genes contribute to disease progression through diverse mechanisms. POLQ, which promotes cell proliferation and migration in HCC, is associated with tumor malignancy and poor prognosis 23. Similarly, TOMM40L, a component of the TOM complex, likely supports cancer progression by enhancing mitochondrial function, akin to TOMM20's role in promoting cell proliferation, migration, and invasion in cancer 24. In addition, DHRS1 plays a significant role in steroid and xenobiotic metabolism 25. Mutations in AASS can lead to impaired lysine degradation, resulting in hyperlysinemia and associated neurological symptoms 26. LDHD is essential for catalyzing the oxidation of d-lactate to pyruvate, regulating d-lactate levels and influencing mitochondrial function and metabolic balance 27. GCDH is critical for melanoma cell survival and proliferation through the regulation of metabolic and apoptotic pathways 28. Conversely, lower GRHPR expression levels in tumors are associated with increased tumor cell proliferation and shorter patient survival 29. ALAS1 is regulated by estrogen and succinate and participates in the control of the proliferation and invasiveness of uterine endometrial cancer 30. In HCC, OGDHL regulates glutamine metabolic pathways and lipid synthesis, significantly affecting tumor cell growth and the response to chemotherapy 31. HMGCS2 plays a crucial role in HCC by modulating ketogenesis. Its downregulation promotes tumor growth and progression, whereas its overexpression inhibits these oncogenic processes 32.

The constructed RDS was based on the expression levels of three mitochondrial genes associated with the TACE NR diagnosis. These genes also have significant implications in disease progression. OGDHL regulates HCC metastasis by modulating HIF-1α activity and stabilization, thereby affecting cell invasiveness and migration 33. MSRA, which is located on chromosome 8p, acts as a metastatic suppressor in HCC by inhibiting cell proliferation and invasion 34. ACSM2A plays a vital role in the liver by catalyzing the activation of medium-chain fatty acids and its genetic variations may influence disease susceptibility 35.

Research has shown that tumor cells can survive in the tumor microenvironment, allowing them to evade immune surveillance and resist drug treatments 36. This study compared the tumor microenvironment between TACE NR and R patients, identifying significant differences in immune cell infiltration. Group R displayed higher levels of CD8+ T cells, NK cells, M1 macrophages, dendritic cells, and B cells, suggesting a strong cytotoxic and proinflammatory immune response. In contrast, the NR group showed reduced infiltration, indicating a less active immune microenvironment. Correlation analysis revealed that a higher RDS was associated with lower levels of cytotoxic immune cells and was negatively correlated with monocytes and M1 macrophages, indicating a less favorable immune environment. Additionally, RDS positively correlated with M0 macrophages and activated mast cells, suggesting increased immunosuppression and inflammation with a higher RDS. Notably, RDS was strongly and negatively correlated with hepatocytes, suggesting a decrease in the number of hepatocytes with a higher RDS. This may explain the lower TACE NR rate in patients with a low RDS, as a lower RDS could be linked to higher immune cell infiltration and a more effective antitumor immune response.

Our study aimed to investigate the differences between high and low RDS and explore alternative therapies for TACE NR. Analysis of the mutation profiles indicated that CTNNB1 was the most commonly mutated gene in patients with a low RDS. Studies have highlighted that CTNNB1 mutations in HCC are correlated with positive clinicopathological characteristics and enhanced survival rates. A meta-analysis of 17 studies involving 1828 patients demonstrated that HCC patients with CTNNB1 mutations exhibited significantly improved 1-, 3-, and 5-year overall survival rates. Moreover, these mutations have been linked to better tumor differentiation, earlier TNM stages, lower prevalence of liver cirrhosis, and reduced HBV infection rates 37. These findings suggest that a low RDS is associated with a favorable prognosis and a high prevalence of CTNNB1 mutations play a crucial role.

In recent years, immunotherapy for HCC has seen advancements in the use of immune checkpoint inhibitors such as PD-1, PD-L1, and CTLA-4 antibodies, offering new hope for treatment 38. Our study found that patients with a high RDS tended to have lower immune response rates. The TIDE score evaluates immune escape and immunosuppression in the tumor microenvironment, and predicts immune checkpoint inhibitor responses. Higher TIDE scores were associated with poorer responses to immunotherapy, and there was a positive correlation between RDS and TIDE scores, indicating that patients with a high RDS tended to have higher TIDE scores. The IPS evaluates tumor immunogenicity and potential immune escape mechanisms to predict the efficacy of immunotherapy. A higher IPS suggests stronger immune activity within the tumor and a potentially better response to immune checkpoint inhibitors. Interestingly, our study found that patients with a high RDS had a significantly lower IPS than those with a low RDS, suggesting that poor outcomes in patients with a high RDS may be attributed to tumor immunosuppression and immune escape mechanisms.

Patients with a low RDS may show improved response rates to both TACE and immunotherapy, whereas patients with a high RDS may not respond well to either treatment. Therefore, this study investigated the potential applications of 198 targeted/chemotherapeutic drugs for HCC. This study aimed to identify alternative drugs for patients with a high RDS and more effective treatment options for patients with a low RDS. A total of 33 valuable drugs were identified. Based on the variances in IC50 values between the high- and low- RDS groups, three responsive drugs were chosen for each group. Paclitaxel, dasatinib, and vincristine are potential alternative therapies for patients with a high RDS, whereas JAK1, rapamycin, and AZD2014 are considered candidate drugs for treating patients with a low RDS. Additionally, a correlation analysis between drug targets and RDS genes suggested that ACSM2A may play a significant role in drug sensitivity.

The application of single-cell sequencing in HCC focuses on uncovering tumor heterogeneity, identifying rare cell subpopulations, and analyzing the immune microenvironment. Single-cell RNA sequencing allows researchers to map the transcriptomic landscape of tumor cells, detect specific gene expression patterns, and identify potential therapeutic targets. These technologies provide valuable insights for precision medicine in HCC, leading to the development of more effective diagnostic and therapeutic approaches 39, 40. Subsequently, we examined the functional significance of RDS genes using single-cell RNA sequencing datasets. By constructing single-cell trajectories, we elucidated the relationship between alterations in gene expression and cell fate. Our hypothesis suggests that most shifts in cell states correspond to specific changes in gene expression, shedding light on how certain genes influence cellular behavior and fate during tumor progression. This analysis deepens our understanding of the molecular mechanisms underlying HCC and may uncover novel therapeutic targets and biomarkers. Examination of cell trajectory and pseudotime data revealed a differentiation process from normal hepatocytes to HCC, accompanied by fluctuations in RDS gene expression levels. Notably, ACSM2A showed a consistent down regulation trend during differentiation, with significantly reduced expression in HCC. These findings imply a critical role for ACSM2A in HCC differentiation. ROC and KM curves further underscored the significance of ACSM2A in tumor diagnosis and prognosis. Validation of RDS gene expression at the cellular level indicated decreased expression in HCC cells, suggesting its potential as a key protective factor.

This study is limited by the use of publicly available datasets, such as TCGA, GEO and ICGC, which may not fully represent the etiological diversity of HCC across different geographic regions. The datasets predominantly focus on specific etiologies, potentially underrepresenting variations such as hepatitis B or C-related HCC and non-alcoholic steatohepatitis related HCC. Consequently, the generalizability of the RPS and RDS models to more diverse patient populations is restricted. To address this, future studies should seek validation in larger, multi-ethnic clinical cohorts to ensure the robustness and broad applicability of these models across diverse HCC etiologies. Furthermore, the current study is constrained by a relatively small sample size for model training, which may limit the statistical power and predictive accuracy of the RPS and RDS. Increasing the sample size in future investigations will enhance the models' capacity to capture the full spectrum of disease variability. Additionally, exploring supplementary biomarkers to refine the scoring systems, and performing experimental validation of the functional roles of the RPS and RDS genes, will be critical steps to enhance their clinical utility and translational potential.

Overall, the RPS and RDS systems are crucial for prognostic evaluation and treatment decision-making in patients with HCC and have wide-ranging clinical applications. Therefore, it is important to pay special attention to patients with high RPS levels in clinical practice. Patients with a high RDS may benefit from treatments, such as paclitaxel, dasatinib, and vincristine, whereas patients with a low RDS can explore different treatment options, such as TACE, immunotherapy, and pharmacotherapy, to determine the most effective approach. The implementation of these systems is expected to enhance the clinical outcomes of patients with HCC and advance personalized therapy. Furthermore, this study highlights ACSM2A as a gene that plays a significant role in drug sensitivity and differentiation of hepatocytes into HCC, making it a potential therapeutic target.

## Supplementary material

The supplementary materials include the following sections: Figure S1: Hierarchical clustering; Figure S2: Comparison of the performance of nomograms; Figure S3: Enrichment analysis; Figure S4: Mutation waterfall plot; R script; Data.

## Figures and Tables

**Table 1:** Primer sequences used for real-time PCR analysis

Gene	Forward primer (5′-3′)	Reverse primer (5′-3′)
ACSM2A (human)	GAGGACTTGGCAGGCTGG	CCTGCTGGCTGTTTTCACTC
MSRA (human)	TCCTCCTCCACAGCCTCTTT	TTGACATGATGTTTGGCCGC
OGDHL (human)	GCCCGCCCGAATGAGTC	GGTTTTCCAACCAGGCGAAG
β-actin (human)	GCTTCTCCTTAATGTCACGC	CCCACACTGTGCCCATCTAC

**Table 2:** TCGA-LIHC patient data

Covariates	Type	All	Test	Train	Pvalue
Censor	Alive	217(63.82%)	69(67.65%)	148(62.18%)	0.4024
	Dead	123(36.18%)	33(32.35%)	90(37.82%)	
Stage	Stage I	161(47.35%)	56(54.9%)	105(44.12%)	0.1668
	Stage II	77(22.65%)	21(20.59%)	56(23.53%)	
	Stage III	78(22.94%)	17(16.67%)	61(25.63%)	
	Stage IV	3(0.88%)	0(0%)	3(1.26%)	
	Unknown	21(6.18%)	8(7.84%)	13(5.46%)	
Tstage	T1	168(49.41%)	58(56.86%)	110(46.22%)	0.1893
	T2	84(24.71%)	25(24.51%)	59(24.79%)	
	T3	72(21.18%)	17(16.67%)	55(23.11%)	
	T4	13(3.82%)	1(0.98%)	12(5.04%)	
	Unknown	3(0.88%)	1(0.98%)	2(0.84%)	
Nstage	N0	237(69.71%)	67(65.69%)	170(71.43%)	0.2449
	N1	3(0.88%)	0(0%)	3(1.26%)	
	Unknown	100(29.41%)	35(34.31%)	65(27.31%)	
Mstage	M0	243(71.47%)	73(71.57%)	170(71.43%)	0.5173
	M1	3(0.88%)	0(0%)	3(1.26%)	
	Unknown	94(27.65%)	29(28.43%)	65(27.31%)	
Gender	Female	108(31.76%)	31(30.39%)	77(32.35%)	0.819
	Male	232(68.24%)	71(69.61%)	161(67.65%)	
Age	Old	133(39.12%)	49(48.04%)	84(35.29%)	0.0658
	Young	205(60.29%)	52(50.98%)	153(64.29%)	
	Unknown	2(0.59%)	1(0.98%)	1(0.42%)	

**Table 3:** GSE104580 patient data

Covariates	Type	All	Test	Train	Pvalue
TACE	Non-response	66(44.9%)	22(48.89%)	44(43.14%)	0.641
	Response	81(55.1%)	23(51.11%)	58(56.86%)	
