# Supplementary material for: Mitochondrial Gene Scoring System: Predicting Prognosis and Precision Therapy for TACE Non-Response in Hepatocellular Carcinoma Patients
Source: J Cancer. 2025 Jan 1;16(2):629–47. doi: 10.7150/jca.103946 (PMC11685679; doi:10.7150/jca.103946)
Supplement: Supplementary file 1 — The supplementary materials include the following sections: Figure S1: Hierarchical clustering; Figure S2: Comparison of the performance of nomograms; Figure S3: Enrichment analysis; Figure S4: Mutation waterfall plot; R script; Data. [file jcav16p0629s1.zip › Figure S1-4 & R script.docx]

**Supplementary figure**


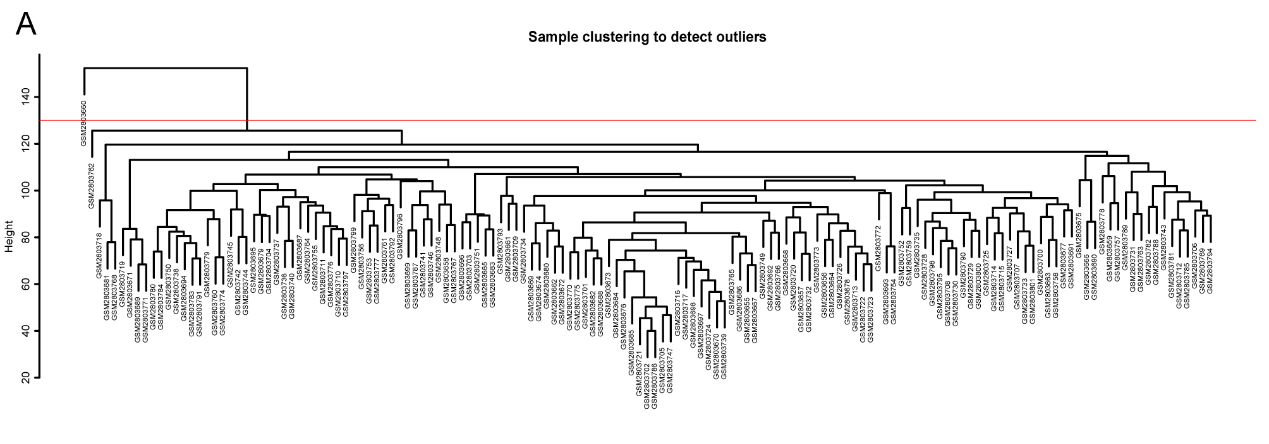
**Fig S1.** Hierarchical clustering of the remaining samples after removing the outlier HCC samples


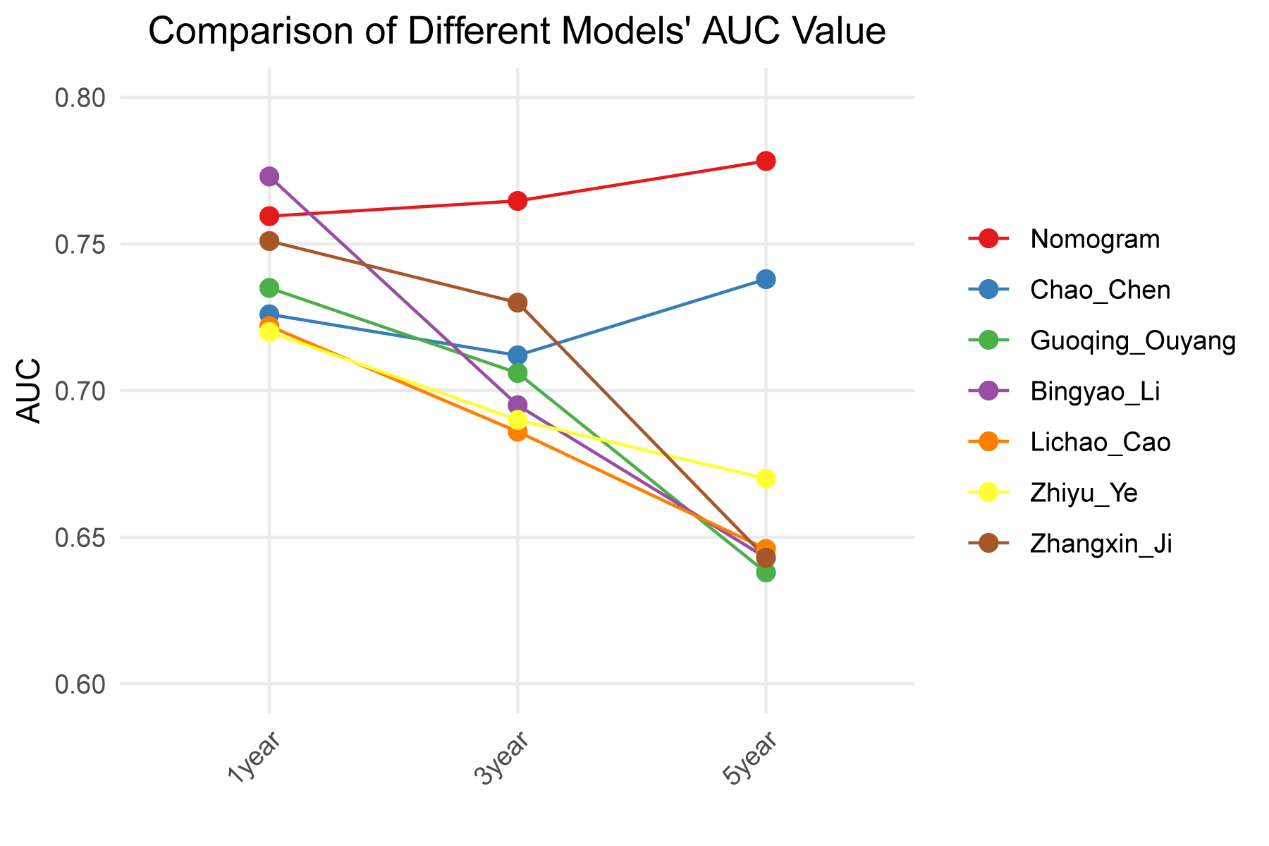


**Fig S2.** The nomogram established in this study was compared with those developed by other researchers


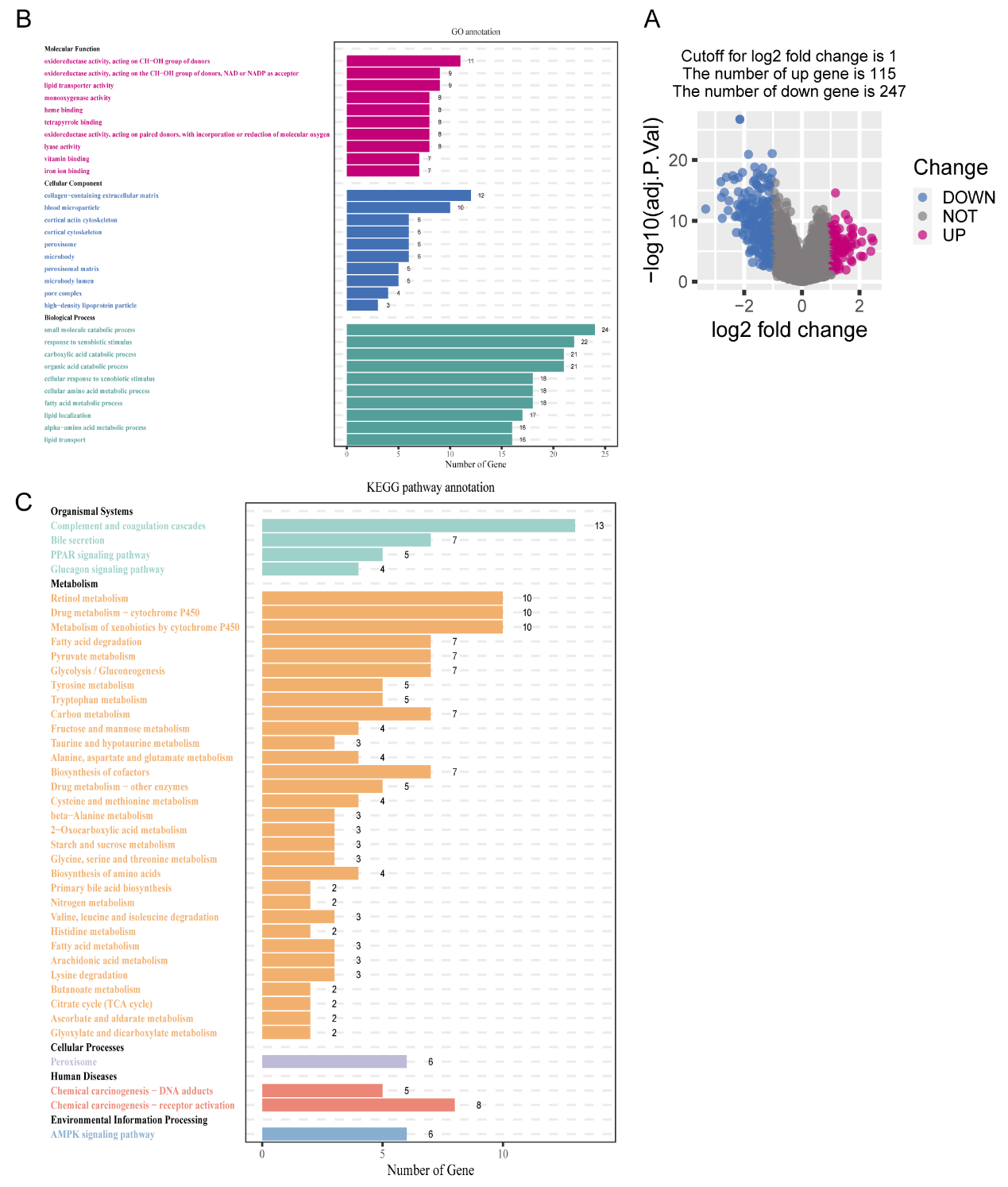


**Fig S3.** Investigating the functional differences between high- and low- RDS groups. (A) Volcano plot of DEGs between high RDS and low RDS. (B) GO enrichment analysis and (C) KEGG enrichment analysis of DEGs between high- and low- RDS groups


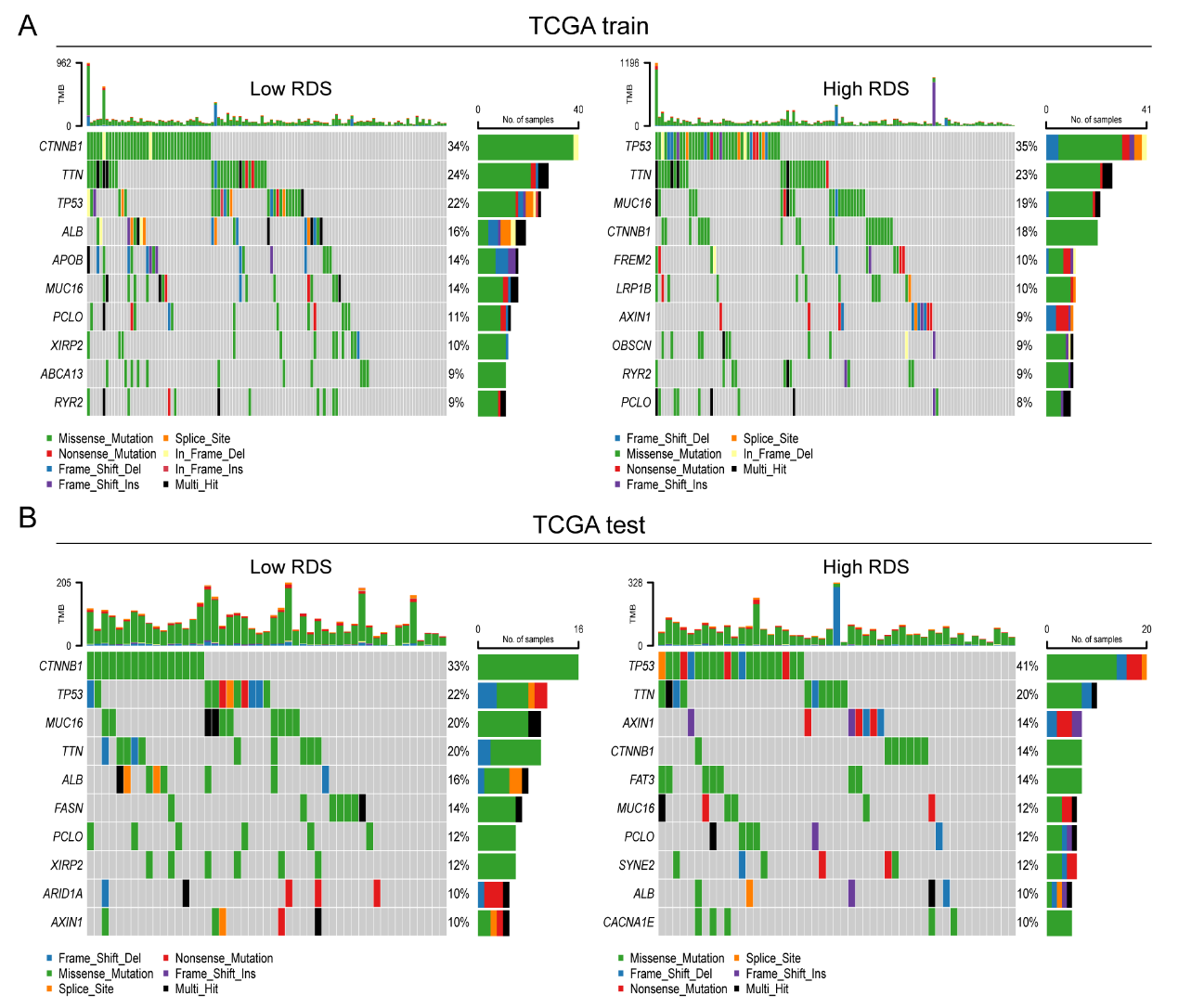


**Fig S4.** (A, B) Mutation waterfall plot between high- and low- RDS groups

**R script**

**WGCNA:**

rm(list = ls())

library(WGCNA)

library(tibble)

mydata<-read.delim("GSE104580exp_average.txt",row.names = 1)

datExpr0 = data.frame(t(mydata))

colnames(datExpr0) <- rownames(mydata)

rownames(datExpr0) <- colnames(mydata)

#Select the top 25% of genes based on variance

datExpr1<-datExpr0

m.vars=apply(datExpr0,2,var)

expro.upper=datExpr0[,which(m.vars>quantile(m.vars, probs = seq(0, 1, 0.25))[4])]

datExpr1<-data.matrix(expro.upper)

gsg = goodSamplesGenes(datExpr1, verbose = 3);

gsg$allOK

if (!gsg$allOK){

# Optionally, print the gene and sample names that were removed:

if (sum(!gsg$goodGenes)>0)

printFlush(paste("Removing genes:", paste(names(datExpr0)[!gsg$goodGenes], collapse = ", ")));

if (sum(!gsg$goodSamples)>0)

printFlush(paste("Removing samples:", paste(rownames(datExpr1)[!gsg$goodSamples], collapse = ", ")));

# Remove the offending genes and samples from the data:

datExpr1 = datExpr1[gsg$goodSamples, gsg$goodGenes]

}

sampleTree = hclust(dist(datExpr1), method = "average")

par(cex = 0.7);

par(mar = c(0,4,2,0))

plot(sampleTree, main = "Sample clustering to detect outliers", sub="", xlab="", cex.lab = 1.5,

cex.axis = 1.5, cex.main = 2)

plot(sampleTree, main = "Sample clustering to detect outliers", sub="", xlab="", cex.lab = 1.5, cex.axis = 1.5, cex.main = 2) +

abline(h = 130, col = "red")

clust = cutreeStatic(sampleTree, cutHeight = 130, minSize = 10)

keepSamples = (clust==1)

datExpr = datExpr1[keepSamples, ]

nGenes = ncol(datExpr)

nSamples = nrow(datExpr)

dim(datExpr)

datExpr <- as.data.frame(datExpr)

#If there is no need to remove outliers, run the following code

#datExpr = as.data.frame(datExpr1)

#nGenes = ncol(datExpr)

#nSamples = nrow(datExpr)

powers = c(c(1:10), seq(from = 12, to=20, by=2))

sft = pickSoftThreshold(datExpr, powerVector = powers, verbose = 5)

pdf("1Threshold.pdf",width = 10, height = 5)

par(mfrow = c(1,2))

cex1 = 0.9

plot(sft$fitIndices[,1], -sign(sft$fitIndices[,3])*sft$fitIndices[,2],

xlab="Soft Threshold (power)",ylab="Scale Free Topology Model Fit,signed R^2",type="n",

main = paste("Scale independence")) +

text(sft$fitIndices[,1], -sign(sft$fitIndices[,3])*sft$fitIndices[,2],

labels=powers,cex=cex1,col="red")+

abline(h=0.90,col="red")

plot(sft$fitIndices[,1], sft$fitIndices[,5],

xlab="Soft Threshold (power)",ylab="Mean Connectivity", type="n",

main = paste("Mean connectivity")) +

text(sft$fitIndices[,1], sft$fitIndices[,5], labels=powers, cex=cex1,col="red")

dev.off()

net = blockwiseModules(datExpr, power = 4,

TOMType = "unsigned", minModuleSize = 30,

reassignThreshold = 0, mergeCutHeight = 0.25,

numericLabels = TRUE, pamRespectsDendro = FALSE,

saveTOMs = TRUE,

#saveTOMFileBase = "MyTOM",

verbose = 3)

table(net$colors)

mergedColors = labels2colors(net$colors)

pdf("2module.pdf",width = 10, height = 5)

plotDendroAndColors(net$dendrograms[[1]], mergedColors[net$blockGenes[[1]]], "Module colors",

dendroLabels = FALSE, hang = 0.03,

addGuide = TRUE, guideHang = 0.05)

dev.off()

moduleLabels = net$colors

moduleColors = labels2colors(net$colors)

MEs = net$MEs;

geneTree = net$dendrograms[[1]]

text <- unique(moduleColors)

for (i in 1:length(text)) {

y=t(assign(paste(text[i],"expr",sep = "."),datExpr[moduleColors==text[i]]))

write.csv(y,paste(text[i],"csv",sep = "."),quote = F)

}

samples=read.delim('GSE104580_feature.txt',row.names = 1)

samples=samples[rownames(datExpr),,drop = FALSE]

identical(rownames(samples),rownames(datExpr))

moduleLabelsAutomatic = net$colors

moduleColorsAutomatic = labels2colors(moduleLabelsAutomatic)

moduleColorsWW = moduleColorsAutomatic

MEs0 = moduleEigengenes(datExpr, moduleColorsWW)$eigengenes

MEsWW = orderMEs(MEs0)

modTraitCor = cor(MEsWW, samples, use = "p")

colnames(MEsWW)

modlues=MEsWW

modTraitP = corPvalueStudent(modTraitCor, nSamples)

textMatrix = paste(signif(modTraitCor, 2), "\n(", signif(modTraitP, 1), ")", sep = "")

dim(textMatrix) = dim(modTraitCor)

pdf("3Module-trait.pdf",width = 4, height = 6)

labeledHeatmap(Matrix = modTraitCor, xLabels = colnames(samples), yLabels = names(MEsWW), cex.lab = 0.5, yColorWidth=0.01,

xColorWidth = 0.03,

ySymbols = colnames(modlues), colorLabels = FALSE, colors = blueWhiteRed(50),

textMatrix = textMatrix, setStdMargins = FALSE, cex.text = 0.5, zlim = c(-1,1)

, main = paste("Module-trait relationships"))

dev.off()

**risk prognosis score:**

rm(list = ls())

library(survival)

library(survminer)

library(tidyverse)

library(glmnet)

library(ggplot2)

library(ggpubr)

library(timeROC)

library(pROC)

min_max_scale = function(x){

(x-min(x))/(max(x)-min(x))}

exp=read.table("TCGA-LIHC-congene_tpm_mRNA_clini.txt", header=T, sep="\t", check.names=F,row.names = 1)

set.seed(123456)

train_id <- sample(1:nrow(exp),0.7*nrow(exp))

train_exp <- exp[train_id,]

test_exp<- exp[-train_id,]

test_exp <- cbind(patientID=rownames(test_exp),test_exp)

# write.table(test_exp,"TCGA-LIHC_test_exp.txt",sep = "\t",quote = F,row.names = F)

exp1 <- train_exp

exp1=as.data.frame(t(exp1))

qx <- as.numeric(quantile(exp1, c(0., 0.25, 0.5, 0.75, 0.99, 1.0), na.rm=T))

LogC <- (qx[5] > 100) ||

(qx[6]-qx[1] > 50 && qx[2] > 0)||

(qx[2] > 0 && qx[2] < 1 && qx[4] > 1 && qx[4] < 2)

LogC

if (LogC) {

exp1 <- log2(exp1+1)

print("log2(exp1+1) transform is finished")

} else {

print("log2(exp1+1) transform is not needed")

}

exp1=as.data.frame(t(exp1))

dimnames=list(rownames(exp1), colnames(exp1))

exp1=matrix(as.numeric(as.matrix(exp1)), nrow=nrow(exp1), dimnames=dimnames)

cli <- read.delim("TCGA-LIHC_Clinical.txt", header=T, sep="\t", check.names=F, row.names=1)

cli <- cli[,c("futime","fustat")]

cli <- cli %>% filter(futime > 1/12)

model_gene=read.table("venn.txt", header=T, sep="\t", check.names=F)

exp1 = exp1[,model_gene$SYMBOL]

sameSample=intersect(row.names(exp1), row.names(cli))

exp1=exp1[sameSample,]

cli=cli[sameSample,]

rt=cbind(cli, exp1)

p.value = 0.05

uniCox_outTab=data.frame()

for(i in colnames(rt[,3:ncol(rt)])){

cox <- coxph(Surv(futime, fustat) ~ rt[,i], data = rt)

coxSummary = summary(cox)

coxP=coxSummary$coefficients[,"Pr(>|z|)"]

if(coxP < p.value){

uniCox_outTab=rbind(uniCox_outTab,

cbind(id=i,

HR=coxSummary$conf.int[,"exp(coef)"],

HR.95L=coxSummary$conf.int[,"lower .95"],

HR.95H=coxSummary$conf.int[,"upper .95"],

pvalue=coxSummary$coefficients[,"Pr(>|z|)"])

)

}

}

write.table(uniCox_outTab, file="uniCox.txt", sep="\t", row.names=F, quote=F)

rt1 <- rt[,c("futime","fustat",uniCox_outTab$id)]

x=as.matrix(rt1[,c(3:ncol(rt1))])

y=data.matrix(Surv(rt1$futime, rt1$fustat))

fit<- glmnet(x,y,family = "cox",alpha = 1,nlambda = 100)

p <- plot(fit,xvar = "lambda",label = F)

ggsave(p,filename="LassoCox1.pdf",wi=4,he=3)

set.seed(123456)

cvfit <- cv.glmnet(x,y,family = "cox",alpha = 1,nlambda = 100)

plot(cvfit)

coef_lambda.min=coef(fit,s=cvfit$lambda.min)

actcoef_lambda.min=coef_lambda.min[which(coef_lambda.min!=0)]

lassoGene=row.names(coef_lambda.min)[which(coef_lambda.min!=0)]

lasso_score=function(x){crossprod(as.numeric(x),actcoef_lambda.min)}

lassoscore=apply(rt1[,lassoGene],1,lasso_score)

lassoscore=min_max_scale(lassoscore)

lassoSigExp=cbind(rt1[,c("futime","fustat",lassoGene)],LASSOscore=lassoscore)

lassoSigExp$LASSOgroup=ifelse(lassoSigExp$LASSOscore>median(lassoSigExp$LASSOscore),"high","low")

lassoSigExp=cbind(patientID=rownames(lassoSigExp),lassoSigExp)

write.table(lassoSigExp,"lassoSigExp.txt",sep = "\t",quote = F,row.names = F)

lasso_outTab=cbind(coef=coef_lambda.min[,1][coef_lambda.min[,1]!=0],HR=exp(coef_lambda.min[,1][coef_lambda.min[,1]!=0]))

lasso_outTab=cbind(id=rownames(lasso_outTab),lasso_outTab)

write.table(lasso_outTab,file="lassoCox.txt",sep="\t",row.names=F,quote=F)

model_result <- read.delim("lassoSigExp.txt",row.names = 1)

KMfit=survfit(Surv(futime,fustat)~LASSOgroup,data = model_result)

pdf("tcga_train_km.pdf",wi=3.5,he=3.5)

ggsurvplot(KMfit,

data = model_result,

surv.median.line = "none",

size = 0.5,

cex.lab=2,

break.x.by = 1,

axis.title.x =element_text(size=5),

axis.title.y = element_text(size=5),

palette = c(High = "#C1565E", Low = "#7EA4D1"),

conf.int = F,

pval = TRUE,

risk.table = TRUE,

xlab = "Overall Survival (years)",

ylab="Survival probability ",

legend.title="RPS",

legend=c(0.85,0.8),

risk.table.col = "strata",# Risk table color by groups

legend.labs = c("High","Low"), # Change legend labels

risk.table.height = 0.35, # Useful to change when you have multiple groups

ggtheme = theme_grey()+ # Change ggplot2 theme

theme(legend.background = element_blank())

)

dev.off()

pdf("tcga_train_ROC.pdf",wi=3.5,he=3.5)

timeROC_res=timeROC(T = model_result$futime,

delta = model_result$fustat,

marker = model_result$LASSOscore,

cause = 1,

weighting="marginal",

times = c(1, 2, 3 ,4 ,5),ROC = TRUE,iid = TRUE)

timeROC_df=data.frame(time = rep(c(1,2,3,4,5), each=nrow(timeROC_res$FP)),

FP = c(timeROC_res$FP[,1], timeROC_res$FP[,2], timeROC_res$FP[,3], timeROC_res$FP[,4], timeROC_res$FP[,5]),

TP = c(timeROC_res$TP[,1], timeROC_res$TP[,2], timeROC_res$TP[,3], timeROC_res$TP[,4], timeROC_res$TP[,5]))

ggplot(data = timeROC_df)+

geom_line(aes(x = FP, y = TP, color = factor(time)), size = 0.7)+

scale_color_manual(values = c("1" = "#807C7D", "2" = "#C1565E", "3" = "#DCA96A", "4" = "#82AD7F", "5" = "#7EA4D1"))+

geom_abline(slope = 1, intercept = 0, color = "black", size = 0.7, linetype = 2)+

theme_grey()+theme(legend.position = "none")+

labs(x = "False positive rate", y = "True positive rate")+

annotate("text",x = 0.7, y = 0.25, size = 3.5,

label = paste0("AUC at 1 years = ", sprintf("%.3f", timeROC_res$AUC[[1]])), color = "#807C7D")+

annotate("text",x = 0.7, y = 0.2, size = 3.5,

label = paste0("AUC at 2 years = ", sprintf("%.3f", timeROC_res$AUC[[2]])), color = "#C1565E")+

annotate("text",x = 0.7, y = 0.15, size = 3.5,

label = paste0("AUC at 3 years = ", sprintf("%.3f", timeROC_res$AUC[[3]])), color = "#DCA96A")+

annotate("text",x = 0.7, y = 0.1, size = 3.5,

label = paste0("AUC at 4 years = ", sprintf("%.3f", timeROC_res$AUC[[4]])), color = "#82AD7F")+

annotate("text",x = 0.7, y = 0.05, size = 3.5,

label = paste0("AUC at 5 years = ", sprintf("%.3f", timeROC_res$AUC[[5]])), color = "#7EA4D1")

dev.off()

**risk diagnosis score:**

rm(list = ls())

library(survival)

library(survminer)

library(tidyverse)

library(glmnet)

library(ggplot2)

library(ggpubr)

library(pROC)

library(rms)

library(plyr)

min_max_scale = function(x){

(x-min(x))/(max(x)-min(x))}

exp=read.table("GSE104580exp_average.txt", header=T, sep="\t", check.names=F,row.names = 1)

exp=t(exp)

set.seed(123456)

train_id <- sample(1:nrow(exp),0.7*nrow(exp))

train_exp <- exp[train_id,]

test_exp<- exp[-train_id,]

test_exp <- cbind(patientID=rownames(test_exp),test_exp)

# write.table(test_exp,"GSE104580test_exp.txt",sep = "\t",quote = F,row.names = F)

exp1 <- as.data.frame(t(train_exp))

qx <- as.numeric(quantile(exp1, c(0., 0.25, 0.5, 0.75, 0.99, 1.0), na.rm=T))

LogC <- (qx[5] > 100) ||

(qx[6]-qx[1] > 50 && qx[2] > 0)||

(qx[2] > 0 && qx[2] < 1 && qx[4] > 1 && qx[4] < 2)

LogC

if (LogC) {

exp1 <- log2(exp1+1)

print("log2(exp1+1) transform is finished")

} else {

print("log2(exp1+1) transform is not needed")

}

exp1=as.data.frame(t(exp1))

dimnames=list(rownames(exp1), colnames(exp1))

exp1=matrix(as.numeric(as.matrix(exp1)), nrow=nrow(exp1), dimnames=dimnames)

cli <- read.delim("GSE104580_clin.txt", header=T, sep="\t", check.names=F, row.names=1)

model_gene=read.table("venn.txt", header=T, sep="\t", check.names=F)

exp1 = exp1[,model_gene$SYMBOL]

sameSample=intersect(row.names(exp1), row.names(cli))

exp1=exp1[sameSample,]

cli=cli[sameSample,]

rt=cbind(diagnosis=cli, exp1)

rt=as.data.frame(rt)

p.value = 0.05

uniLogi=function(x){

FML=as.formula(paste0("diagnosis~",x))

glm1=glm(FML,data = rt,family = "binomial")

glm2=summary(glm1)

OR=exp(coef(glm1))

SE=glm2$coefficients[,2]

CI5=exp(coef(glm1)-1.96*SE)

CI95=exp(coef(glm1)+1.96*SE)

Pvalue=glm2$coefficients[,4]

uniLogi=data.frame("id"=x,

"OR"=OR,

"CI5"=CI5,

"CI95"=CI95,

"Pvalue"=Pvalue)[-1,]

return(uniLogi)

}

uniLog=lapply(t(model_gene),uniLogi)

uniLog=ldply(uniLog,data.frame)

uniLog=subset(uniLog,Pvalue<p.value)

write.table(uniLog, file="uniLog.txt", sep="\t", row.names=F, quote=F)

rt1 <- rt[,c("diagnosis",uniLog$id)]

x=as.matrix(rt1[,c(2:ncol(rt1))])

y=as.matrix(rt1[,1])

set.seed(123456)

fit<- glmnet(x,y,family = "binomial",alpha = 1,nlambda = 100)

plot(fit,xvar = "lambda",label = F)

cvfit <- cv.glmnet(x,y,family = "binomial",alpha = 1,nlambda = 100)

plot(cvfit)

coef_lambda.min=coef(fit,s=cvfit$lambda.min)

actcoef_lambda.min=coef_lambda.min[which(coef_lambda.min!=0)][-1]

lassoGene=row.names(coef_lambda.min)[which(coef_lambda.min!=0)][-1]

lasso_score=function(x){crossprod(as.numeric(x),actcoef_lambda.min)}

lassoscore=apply(rt1[,lassoGene],1,lasso_score)

lassoscore=min_max_scale(lassoscore)

lassoSigExp=cbind(rt1[,c("diagnosis",lassoGene)],LASSOscore=lassoscore)

lassoSigExp$LASSOgroup=ifelse(lassoSigExp$LASSOscore>median(lassoSigExp$LASSOscore),"high","low")

lassoSigExp=cbind(patientID=rownames(lassoSigExp),lassoSigExp)

write.table(lassoSigExp,"lassoSigExp.txt",sep = "\t",quote = F,row.names = F)

lasso_outTab=cbind(coef=coef_lambda.min[,1][coef_lambda.min[,1]!=0][-1],HR=exp(coef_lambda.min[,1][coef_lambda.min[,1]!=0][-1]))

lasso_outTab=cbind(id=rownames(lasso_outTab),lasso_outTab)

write.table(lasso_outTab,file="lassoLogstic.txt",sep="\t",row.names=F,quote=F)

model_result <- read.delim("lassoSigExp.txt",row.names = 1)

pdf("GSE104580_train_roc.pdf",wi=3.5,he=3.5)

ROC_res=roc(model_result$diagnosis,model_result$LASSOscore)

ggroc(ROC_res,alpha = 1,size = 0.7,

legacy.axes = T,color = "#79438E")+

geom_abline(slope = 1, intercept = 0, color = "black", size = 0.7, linetype = 2)+

theme_grey()+

labs(x = "False positive rate", y = "True positive rate")+

annotate("text",x = 0.75, y = 0.2, size = 3.5,

label = paste0("AUC = ", sprintf("%.3f", ROC_res[["auc"]])), color = "#79438E")

dev.off()

**Immune_infiltration:**

rm(list=ls())

library(tidyverse)

library(IOBR)

data=read.delim("GSE104580exp_average.txt",row.names = 1)

### 1.cibersort ### ------------------------------------------------------------

cibersort=deconvo_tme(eset = data, method = "cibersort", arrays = TRUE, perm = 1000)

#write.table(cibersort,"cibersort.txt",sep = "\t",quote = F,row.names = F)

### 2.estimate ### -------------------------------------------------------------

estimate=deconvo_tme(eset = data, method = "estimate")

#write.table(estimate,"estimate.txt",sep = "\t",quote = F,row.names = F)

### 3.epic ### -----------------------------------------------------------------

epic=deconvo_tme(eset = data, method = "epic", arrays = TRUE)

#write.table(epic,"epic.txt",sep = "\t",quote = F,row.names = F)

### 4.mcp_counter ### ----------------------------------------------------------

mcp=deconvo_tme(eset = data, method = "mcpcounter")

#write.table(mcp,"mcp.txt",sep = "\t",quote = F,row.names = F)

### 5.xcell ### ----------------------------------------------------------------

xcell=deconvo_tme(eset = data, method = "xcell", arrays = TRUE)

#write.table(xcell,"xcell.txt",sep = "\t",quote = F,row.names = F)

### 6.timer ### ----------------------------------------------------------------

timer=deconvo_tme(eset = data, method = "timer", group_list = rep("stad",dim(data)[2]))

#write.table(timer,"timer.txt",sep = "\t",quote = F,row.names = F)

### 7.quanTIseq ### ------------------------------------------------------------

quantiseq=deconvo_tme(eset = data, tumor = TRUE, arrays = TRUE, scale_mrna = TRUE, method = "quantiseq")

#write.table(quantiseq,"quantiseq.txt",sep = "\t",quote = F,row.names = F)

### 8.ips ### ------------------------------------------------------------------

ips=deconvo_tme(eset = data, method = "ips", plot= FALSE)

#write.table(ips,"ips.txt",sep = "\t",quote = F,row.names = F)

tme_combine<-cibersort %>%

inner_join(.,mcp,by = "ID") %>%

inner_join(.,xcell,by = "ID") %>%

inner_join(.,epic,by = "ID") %>%

inner_join(.,estimate,by = "ID") %>%

inner_join(.,timer,by = "ID") %>%

inner_join(.,quantiseq,by = "ID") %>%

inner_join(.,ips,by = "ID")

dim(tme_combine)

write.table(tme_combine,"tme_combine.txt",sep = "\t",quote = F,row.names = F)

**drug sensitivity:**

rm(list = ls())

library(oncoPredict)

data=read.table("LIRI-JP_tumor_tpm.txt", header=T, sep="\t", check.names=F,row.names = 1)

#data=as.data.frame(t(data))

dimnames=list(rownames(data),colnames(data))

data=matrix(as.numeric(as.matrix(data)), nrow=nrow(data), dimnames=dimnames)

GDSC2_Expr=readRDS(file='GDSC2_Expr.rds')

GDSC2_Res=readRDS(file = 'GDSC2_Res.rds')

GDSC2_Res=exp(GDSC2_Res)

set.seed(123456)

calcPhenotype(trainingExprData = GDSC2_Expr,

trainingPtype = GDSC2_Res,

testExprData = data,

batchCorrect = 'standardize',

powerTransformPhenotype = TRUE,

removeLowVaryingGenes = 0.2,

minNumSamples = 10,

printOutput = TRUE,

removeLowVaringGenesFrom = 'rawData')

senstivity=read.csv("calcPhenotype_Output/DrugPredictions.csv", header=T, sep=",", check.names=F, row.names=1)

colnames(senstivity)=gsub("(.*)\\_(\\d+)", "\\1", colnames(senstivity))

group=read.delim("icgc_result.txt",row.names = 1)

consam=intersect(rownames(group),rownames(senstivity))

group=group[consam, "Group",drop=F]

senstivity=senstivity[consam,,drop=F]

identical(rownames(group),rownames(senstivity))

senstivity[is.na(senstivity)]=0

senstivity=log2(senstivity+1)

rt=cbind(RDS=group$Group, senstivity)

rt$RDS=factor(rt$RDS, levels=c("Low", "High"))

type=levels(factor(rt[,"RDS"]))

comp=combn(type, 2)

my_comparisons=list()

for(i in 1:ncol(comp)){my_comparisons[[i]]<-comp[,i]}

sigGene=c()

for(i in colnames(rt)[2:(ncol(rt))]){

if(sd(rt[,i])<0.05){next}

wilcoxTest=wilcox.test(rt[,i] ~ rt[,"RDS"])

pvalue=wilcoxTest$p.value

if(wilcoxTest$p.value<0.05){

sigGene=c(sigGene, i)

}

}

sigGene=c("RDS",sigGene)

rt=rt[,sigGene]

rt=cbind(patientID=rownames(rt),rt)

write.table(rt,"sig_drug.txt",sep = "\t",quote = F,row.names = F)

**cell chat:**

rm(list = ls())

Sys.setenv(LANGUAGE="en")

options(future.globals.maxSize= 1000*1024^2)

library(dplyr)

library(Seurat)

library(patchwork)

library(ggplot2)

library(tidyverse)

library(NMF)

library(ggalluvial)

library(CellChat)

pbmc <- readRDS("3pbmc_celltype.rds")

data.input = GetAssayData(pbmc,assay = "RNA",layer = "data")

meta = pbmc@meta.data[,c("celltype"),drop=F]

cellchat <- createCellChat(object = data.input)

cellchat <- addMeta(cellchat, meta = meta)

cellchat <- setIdent(cellchat, ident.use = "celltype")

levels(cellchat@idents)

groupSize <- as.numeric(table(cellchat@idents))

cellchat@DB <- subsetDB(CellChatDB.human)

cellchat <- subsetData(cellchat)

future::plan("multisession", workers = 6)

cellchat <- identifyOverExpressedGenes(cellchat)

cellchat <- identifyOverExpressedInteractions(cellchat)

options(future.globals.maxSize= 1048576000)

cellchat <- computeCommunProb(cellchat,raw.use = T)

cellchat <- filterCommunication(cellchat, min.cells = 10)

cellchat <- computeCommunProbPathway(cellchat)

cellchat <- aggregateNet(cellchat)

df.net <- subsetCommunication(cellchat)

write.table(df.net,"cellchat_data.txt",sep = "\t",quote = F,row.names = F)

groupSize <- as.numeric(table(cellchat@idents))

pdf("9Number of interactions.pdf",wi=5,he=6)

netVisual_circle(cellchat@net$count, vertex.weight = groupSize, weight.scale = T, label.edge= F, title.name = "Number of interactions")

dev.off()

pdf("10Interaction weights strength.pdf",wi=5,he=6)

netVisual_circle(cellchat@net$weight, vertex.weight = groupSize, weight.scale = T, label.edge= F, title.name = "Interaction weights/strength")

dev.off()

i="Hepatocytes"

mat <- cellchat@net$count

mat2 <- matrix(0, nrow = nrow(mat), ncol = ncol(mat), dimnames = dimnames(mat))

mat2[i, ] <- mat[i, ]

pdf("11Hepatocytes Number of interactions.pdf",wi=5,he=6)

netVisual_circle(mat2, vertex.weight = groupSize, weight.scale = T, edge.weight.max = max(mat), title.name = rownames(mat)[i])

dev.off()

mat <- cellchat@net$weight

mat2 <- matrix(0, nrow = nrow(mat), ncol = ncol(mat), dimnames = dimnames(mat))

mat2[i, ] <- mat[i, ]

pdf("12Hepatocytes Interaction weights strength.pdf",wi=5,he=6)

netVisual_circle(mat2, vertex.weight = groupSize, weight.scale = T, edge.weight.max = max(mat), title.name = rownames(mat)[i])

dev.off()

cellchat <- netAnalysis_computeCentrality(cellchat, slot.name = "netP")

selectK(cellchat, pattern = "outgoing")

selectK(cellchat, pattern = "incoming")

nPatterns = 2

cellchat <- identifyCommunicationPatterns(cellchat, pattern = "outgoing", k = nPatterns)

pdf("13outgoing.pdf",wi=13,he=3)

netAnalysis_dot(cellchat, pattern = "outgoing")

dev.off()

nPatterns = 2

cellchat <- identifyCommunicationPatterns(cellchat, pattern = "incoming", k = nPatterns)

pdf("14incoming.pdf",wi=13,he=3)

netAnalysis_dot(cellchat, pattern = "incoming")

dev.off()

saveRDS(cellchat, file = "4cellchat.rds")

**copycat:**

rm(list = ls())

Sys.setenv(LANGUAGE="en")

library(Seurat)

library(copykat)

library(tidyverse)

library(harmony)

pbmc <- readRDS("3pbmc_celltype.rds")

pbmc <- subset(pbmc, idents = "Hepatocytes")

pbmc <- CreateSeuratObject(GetAssayData(pbmc, assay="RNA", layer="counts"), meta.data = pbmc@meta.data)

pbmc <- NormalizeData(pbmc, normalization.method = "LogNormalize", scale.factor = 10000)

pbmc <- FindVariableFeatures(pbmc, selection.method = "vst", nfeatures = 3000)

pbmc <- ScaleData(pbmc)

pbmc <- RunPCA(pbmc, features = VariableFeatures(pbmc))

s.genes <- cc.genes$s.genes

g2m.genes <- cc.genes$g2m.genes

pbmc <- CellCycleScoring(pbmc, s.features = s.genes, g2m.features = g2m.genes, set.ident = TRUE)

pbmc <- RunPCA(pbmc, features = c(s.genes, g2m.genes))

CellCycle <- DimPlot(pbmc,reduction = "pca")

pbmc <- ScaleData(pbmc, vars.to.regress = c("S.Score", "G2M.Score"))

pbmc <- RunPCA(pbmc, features = VariableFeatures(pbmc))

remove_CellCycle <- DimPlot(pbmc, reduction = "pca")

pca <- DimPlot(pbmc,reduction = "pca",group.by = "orig.ident")

pbmc <- RunHarmony(pbmc, group.by.vars = "orig.ident")

harmony <- DimPlot(pbmc,reduction = "harmony",group.by = "orig.ident")

ElbowPlot(pbmc, ndims=50, reduction="harmony")

pbmc <- FindNeighbors(pbmc, reduction = "harmony", dims = 1:25) %>%

FindClusters(resolution = 0.05)

set.seed(123456)

pbmc <- RunUMAP(pbmc, reduction = "harmony", dims = 1:25)

pbmc <- subset(pbmc, idents = 0:2)

p_umap <- DimPlot(pbmc, reduction = "umap", raster=FALSE, label = T, repel = F)

# ggsave(p_umap,filename="15Hepatocytes_umap_cluster.pdf",wi=5.5,he=4)

saveRDS(pbmc,"Hepatocytes_cluster.rds")

counts <- GetAssayData(pbmc, assay="RNA", layer="counts")

cnv <- copykat(rawmat=counts, ngene.chr=5, sam.name="Hepatocytes", n.cores=6)

saveRDS(cnv, "cnv.rds")

pred.test <- data.frame(cnv$prediction)

write.table(pred.test,"pred.test.txt",sep = "\t",quote = F,row.names = F)

pred.test <- read.table("pred.test.txt",sep = "\t",row.names = 1,header = T)

identical(rownames(pred.test),rownames(pbmc@meta.data))

pred.test <- pred.test[rownames(pbmc@meta.data),,drop=F]

identical(rownames(pred.test),rownames(pbmc@meta.data))

pbmc <- AddMetaData(pbmc, metadata = pred.test)

Idents(pbmc) <- pbmc$copykat.pred

pbmc <- subset(pbmc, idents = c("diploid","aneuploid"))

pbmc$tissue <- ifelse(pbmc$copykat.pred=="diploid","NHep","HCC")

p_umap <- DimPlot(pbmc, reduction = "umap", raster=FALSE, label = T, repel = F, group.by = "tissue")

VlnPlot(pbmc,features = "ACSM2A",pt.size = 0)

saveRDS(pbmc, file = "5pbmc_copykat.rds")

**cell trajectory:**

rm(list = ls())

Sys.setenv(LANGUAGE="en")

library(Seurat)

library(tidyverse)

library(SingleCellExperiment)

library(slingshot)

library(formattable)

library(tradeSeq)

library(ggplot2)

pbmc <- readRDS("5pbmc_copykat.rds")

Idents(pbmc) <- pbmc$tissue

sim <- as.SingleCellExperiment(pbmc, assay = "RNA")

sim <- slingshot(sim,

clusterLabels = 'seurat_clusters',

reducedDim = 'UMAP',

start.clus= "2",

end.clus = "1",

reweight = FALSE)

SlingshotDataSet(sim)

cell_colors <- c("HCC"="#e60013","NHep"="grey")

point_colors <- cell_colors[sim$tissue]

plot(reducedDims(sim)$UMAP, pch=16, asp = 1, col=point_colors, bty = "n", xaxt = "n", yaxt = "n", xlab = "", ylab = "", cex = 0.05)

lines(SlingshotDataSet(sim), lwd=1.5, col = "black")

slingsce<-SlingshotDataSet(sim)

pseudotimeED <- slingPseudotime(slingsce, na = FALSE)

cellWeightsED <- slingCurveWeights(slingsce)

var_genes <- VariableFeatures(pbmc)

counts <- sim@assays@data@listData$counts

counts <- counts[var_genes,]

sce_slcountsinghot <- fitGAM(counts = counts, pseudotime = pseudotimeED, cellWeights = cellWeightsED, verbose = T)

saveRDS(sce_slcountsinghot,"6sce_slcountsinghot.rds")

gene_expr <- assays(sim)$counts["OGDHL", ]

plot_data_filtered <- data.frame(

Pseudotime = as.numeric(pseudotimeED[, 1]),

Expression = as.numeric(gene_expr),

CellType = factor(sim$tissue)

)

p <- ggplot(plot_data, aes(x = Pseudotime, y = log2(Expression+1), color = CellType)) +

geom_point() +

geom_smooth(data = plot_data_filtered, aes(x = Pseudotime, y = log2(Expression+1)), se = T, color = 'orange') +

# geom_smooth(se = T, color = 'orange') +

scale_color_manual(values = cell_colors) +

theme_classic() +

labs(x = "Pseudotime",

y = "Expression",

color = "Cell Type") +

theme(panel.border = element_rect(fill=NA,color="black", size=0.5, linetype="solid"))
